# Structural basis and evolutionary pathways of glycerol-1-phosphate transport in marine bacteria

**DOI:** 10.1073/pnas.2524546122

**Published:** 2025-12-09

**Authors:** Ning Wang, Linda M. Westermann, Mingyu Li, Chun-Yang Li, Andrew R. J. Murphy, Zengtian Gu, Eleonora Silvano, Claudia A. Blindauer, Ian D. E. A. Lidbury, Yu-Zhong Zhang, David J. Scanlan, Yin Chen

**Affiliations:** ^a^MOE Key Laboratory of Evolution and Marine Biodiversity, State Key Laboratory of Marine Food Processing and Safety Control, College of Marine Life Sciences & Frontiers Science Center for Deep Ocean Multispheres and Earth System, Ocean University of China, Qingdao 266003, China; ^b^State Key Laboratory of Microbial Technology, Marine Biotechnology Research Center, Shandong University, Qingdao 266237, China; ^c^School of Life Sciences, University of Warwick, Coventry CV4 7AL, United Kingdom; ^d^School of Biosciences, University of Birmingham, Edgbaston B15 2TT, United Kingdom; ^e^Department of Chemistry, University of Warwick, Coventry CV4 7AL, United Kingdom; ^f^Molecular Microbiology: Biochemistry to Disease, School of Biosciences, University of Sheffield, Sheffield S10 2TN, United Kingdom; ^g^Institute of Microbiology and Infection, University of Birmingham, Edgbaston B15 2TT, United Kingdom

**Keywords:** glycerol-1-phosphate, Phaeobacter sp. MED193, marine bacteria, glycerol-3-phosphate

## Abstract

All cells use lipid membranes to maintain cellular integrity and function, though Archaea utilize lipids composed of glycerol-1-phosphate (G1P), while Bacteria and Eukaryotes use glycerol-3-phosphate (G3P). Given that Archaea contribute significantly to global marine biomass, accounting for 0.3 gigatonnes (Gt) of carbon in the oceans, we aimed to uncover how archaeal G1P is recycled by marine microorganisms. Through a multidisciplinary approach combining microbiology, biochemistry, and structural biology, we identified a G1P transporter in marine bacteria, which we named GpxB. Phylogenetic analysis revealed that GpxB belongs to the organic phosphonate transporter (PhnT) family and is widely distributed in the marine microbiome, found in approximately 5 to 10% of microbial cells in surface marine waters. Strikingly, we also identified a second G1P transporter, UgpB, that is known to transport G3P and belongs to the carbohydrate uptake transporter-1 (CUT1) family, in the model bacterium *Phaeobacter* sp. MED193. To explore the evolutionary pathways that led to the formation of G1P binding sites in both the PhnT and CUT1 families, we determined the structures of GpxB and UgpB bound to G1P and G3P. Using structure-guided mutagenesis and a comparative analysis of the binding pockets within the PhnT and CUT1 families, we traced their evolutionary trajectories, highlighting the distinct strategies through which G1P-binding sites developed in these two protein families.

## Significance

Archaea contribute substantially to global marine biomass, yet how their unique membrane building block, glycerol-1-phosphate, is recycled has remained unexplored. This study identifies two distinct bacterial transport systems, GpxB and UgpB, that mediate high-affinity uptake of both glycerol-1-phosphate and glycerol-3-phosphate. These transporters are widespread and actively expressed in marine microbiomes, indicating that glycerol-1-phosphate cycling represents a previously overlooked link between archaeal biomass and the global carbon and phosphorus cycles. In sharp contrast to the key enzymes involved in glycerol-1-phosphate and glycerol-3-phosphate biosynthesis, these transporter proteins show no chiral selectivity and can bind to both compounds with similar affinity. By uncovering the structural and evolutionary basis of glycerol-1-phosphate transport, this work provides key insights into glycerol phosphate cycling in the oceans.

All cells possess lipid membranes to maintain their integrity and provide essential energy generating mechanisms. Across the three domains of life bacteria and eukaryotes contain membranes comprising fatty acids linked to a sn-glycerol-3-phosphate (G3P) backbone via ester bonds, while Archaea possess isoprenoid hydrocarbon chains linked by ether linkages to a sn-glycerol-1-phosphate (G1P) backbone. Such a divergence in lipid composition has prompted much discussion about the lipid composition of the last universal common ancestor (LUCA) and that LUCA possessed a mixed bacterial/archaeal membrane (1, 2). G1P and G3P represent the core structure of all glycerophospholipids across all domains of life, suggesting these molecules are widely available in all environments.

Bacterial utilization of organic phosphorus sources is a widespread strategy to obtain phosphorus (P) for growth, particularly under conditions when inorganic phosphate is limiting (3). Phospholipids are key constituents of the particulate organic carbon (POC) pool in the global ocean, representing 5 to 30% of POC in surface marine waters (4, 5), yet their utilization and catabolism have received relatively little attention (6, 7). Recently, we provided evidence that bacterial phospholipid headgroups are degraded by marine microbes liberating the nitrogen-containing moieties that are transported across the bacterial membrane and catabolized internally to support microbial growth, highlighting how lipid degradation in the oceans directly links the biogeochemical cycling of P, nitrogen (N), and carbon (C) (6, 7). Surprisingly, the turnover of archaeal lipids in natural environments has been poorly studied, as previous research has primarily focused on their detection as molecular proxies for paleoenvironmental reconstruction in sedimentary settings (8). However, early laboratory microcosm experiments and more recent environmental studies of sediment samples have shown that the ether bonds in archaeal lipids can indeed undergo hydrolysis, leading to the release of glycerol monoether lipids and the corresponding long-chain isoprenoid or nonisoprenoid alcohols (9–11).

While there is a general dearth of data on the diversity and abundance of specific lipid classes in oceanic waters (12, 13), how the Archaea-derived G1P is utilized by marine microbes remains unknown. G1P serves as the fundamental building block for all archaeal membrane lipids, and Archaea play a crucial role in the biogeochemical cycles of C and N in the global ocean, particularly in ammonium oxidation and methane cycling (14, 15). Recent estimates suggest that Archaea account for 10^28^ to 10^29^ cells in the global ocean (16, 17) and they alone contribute ~0.3 Gt of marine carbon (18). Given the widespread distribution and abundance of Archaea across Earth’s ecosystems (19), we aimed to identify how G1P, the hallmark backbone of archaeal membrane lipids, is used by marine microorganisms.

Here, we identify an ABC transporter, termed GpxB, which is a member of the phosphonate uptake transporter family (3.A.1.9) and can transport both G1P and G3P with high affinity. Strikingly, the UgpB transporter, belonging to the carbohydrate uptake transporter family (3.A.1.1), can also transport G1P, despite UgpB and GpxB being distinct protein families with no sequence homology. Through crystallization studies, we elucidated the structural basis for G1P transport by these transporters and propose an evolutionary path explaining GpxB and UgpB selectivity for G1P/G3P in their respective transporter families. Thus, our study not only identifies G1P transporters but also reveals two fundamentally different evolutionary pathways for the development of G1P binding by nature.

## Results and Discussion

### Identification of a G1P/G3P Transporter, GpxB, in Diverse Bacterial and Archaeal Taxa.

**Fig. 1.**
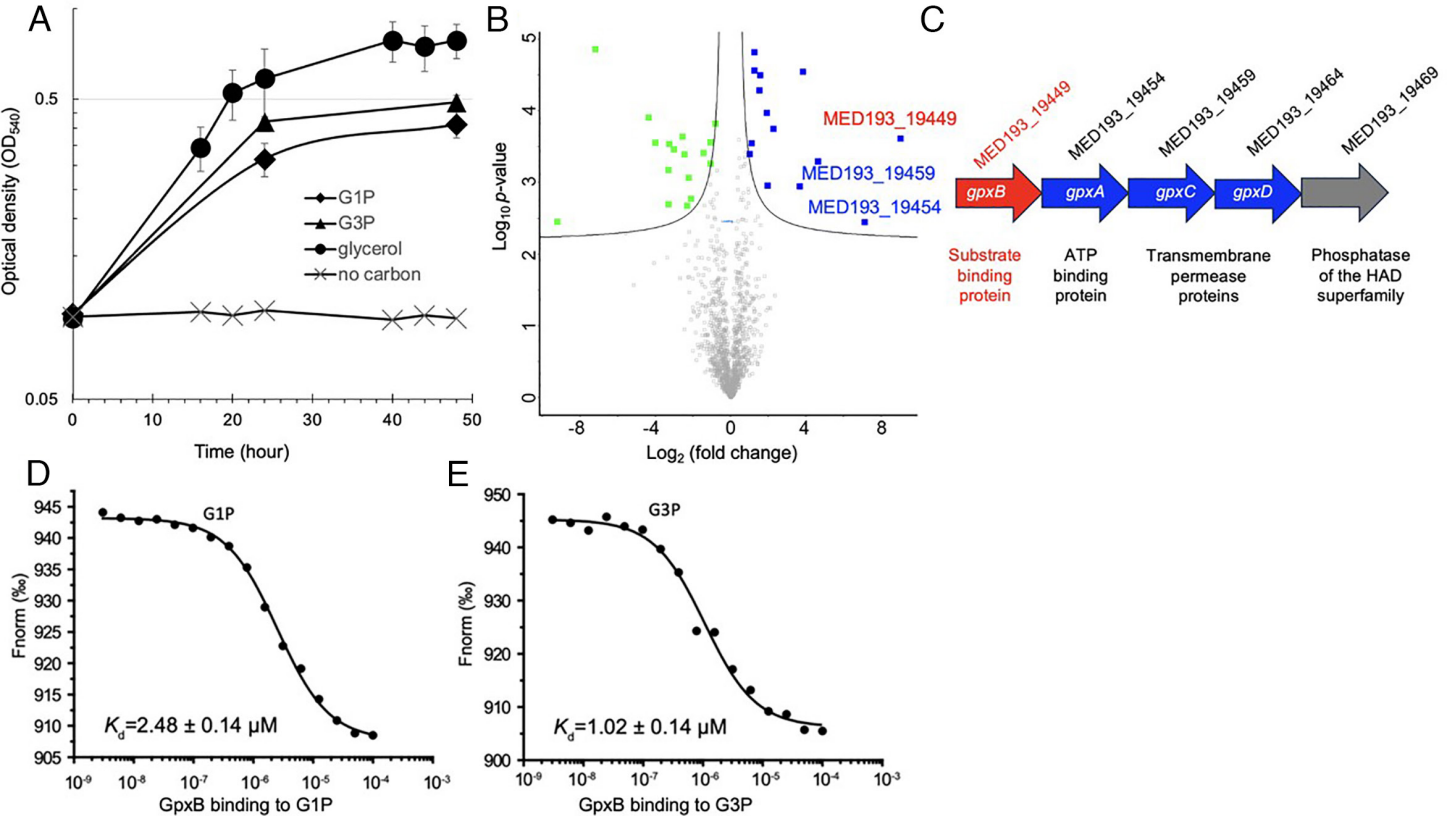
GpxB (MED193_19449) is a G1P binding protein. (*A*) Growth of *Phaeobacter* sp. MED193 on G1P and G3P. *Phaeobacter* sp. MED193 was cultured in defined artificial seawater medium (ASW) supplemented with 5 mM of either G1P (diamond), G3P (triangle), no carbon control (cross), or glycerol (circle) as the positive control. (*B*) Volcano plots of cellular proteomics data comparing *Phaeobacter* sp. MED193 grown on G1P and glycerol as the sole C source. GpxB is highlighted in red. Scatter points represent proteins. The *x*-axis is the fold change for the ratio between both growth conditions and the *y*-axis is the statistical *P*-value. Blue squares represent proteins that are significantly upregulated in the G1P growth condition whereas green squares represent those proteins that are significantly downregulated (*P*-value < 0.05, log2 fold-change). A complete list of differentially regulated proteins by G1P is shown in *SI Appendix*, Table S1. (*C*) Gene neighborhood of the *Phaeobacter* sp. MED193 G1P transporter, including the substrate-binding protein GpxB, the ATP binding protein GpxA and the transmembrane proteins GpxC and GpxD. (*D* and *E*) Detection of G1P (*D*) and G3P (*E*) binding to purified *Phaeobacter* sp. MED193 GpxB by microscale thermophoresis (MST). The change in MST signals was fitted (black lines) to yield a *K_d_* of 2.48 and 1.02 µM for G1P and G3P respectively.

In our ongoing efforts to understand lipid metabolism in *Phaeobacter* sp. MED193, a model strain isolated from oligotrophic Mediterranean Sea surface waters (6, 20), we found that this strain grows on G1P as the sole C source (Fig. 1*A*). It also grows on G3P and glycerol as a carbon source. Thus, *Phaeobacter* sp. MED193 can import and utilize G1P for growth and a G1P transporter must exist in this bacterium. Since a G1P transporter has not been documented before, we conducted comparative proteomics to uncover the mechanism of import and degradation of the lipid backbone moiety G1P. Thus, *Phaeobacter* sp. MED193 was cultured in G1P, G3P, or glycerol, and the cellular proteomes were compared. The addition of G1P (relative to the addition of glycerol) significantly induced the synthesis of 24 proteins (*P*-value < 0.05) in the cellular proteome (*SI Appendix*, Table S1). Noticeably, components of a putative phosphonate ABC transporter showed the greatest fold-change (Fig. 1*B*, *P* < 0.05). The ABC transporter comprised ORFs MED193_19449 (substrate-binding component, hereafter called GpxB), MED193_19454 (ATPase component, GpxC) and MED193_19459 (permease component, GpxD) (Fig. 1*C*). Interestingly, the same ABC transporter system was also highly upregulated when G3P was supplemented (*SI Appendix*, Fig. S1 and Table S1). Thus, we reasoned this transporter is likely capable of importing both G1P and G3P.

To determine the substrate specificity of the periplasmic binding component encoded by MED193_19449 (GpxB) we purified recombinant GpxB protein and undertook affinity assays using microscale thermophoresis (MST). Among the array of compounds tested, recombinant GpxB had the highest binding affinity (*K*_d_) for G1P (*K*_d_ = 2.48 ± 0.14 μM) and G3P (*K*_d_ = 1.02 ± 0.14 μM) (Fig. 1 *D* and *E*). Other 3-carbon (C_3_) phosphate substrates tested showed significantly lower or no binding (*SI Appendix*, Fig. S2), suggesting this transporter is specific for G1P/G3P. The strong binding of GpxB to G1P and G3P appears to be a conserved feature, given that three other GpxB proteins from *Sulfitobacter pseudonitzschiae* DSM26824, *Sulfitobacter* sp. EE-36, and *Sulfitobacter geojensis* EhN01 all exhibited low-µM binding affinity toward G1P/G3P (*SI Appendix*, Fig. S3).

Genome analysis suggests this G1P transporter appears widely distributed in a variety of bacterial and archaeal taxa, noticeably alphaproteobacterial Rhodobacterales, Hyphomicrobiales, Rhodospirillales, gammaproteobacterial Oceanospirillales, Pseudomonadales, Vibrionales, betaproteobacterial Burkholderiales, and the heterotrophic archaeon Haloferacaceae (*SI Appendix*, Fig. S4 and Table S2). Analyses of *gpxB* abundance and transcription in the global Tara Ocean database confirmed that it is widely distributed across various Alphaproteobacteria, actively expressed in global ocean microbiomes, and present in approximately 1 out of every 10 to 20 cells in surface ocean environments (*SI Appendix*, Fig. S5).

### The Bacterial UgpB Transporter Also Binds G1P.

**Fig. 2.**
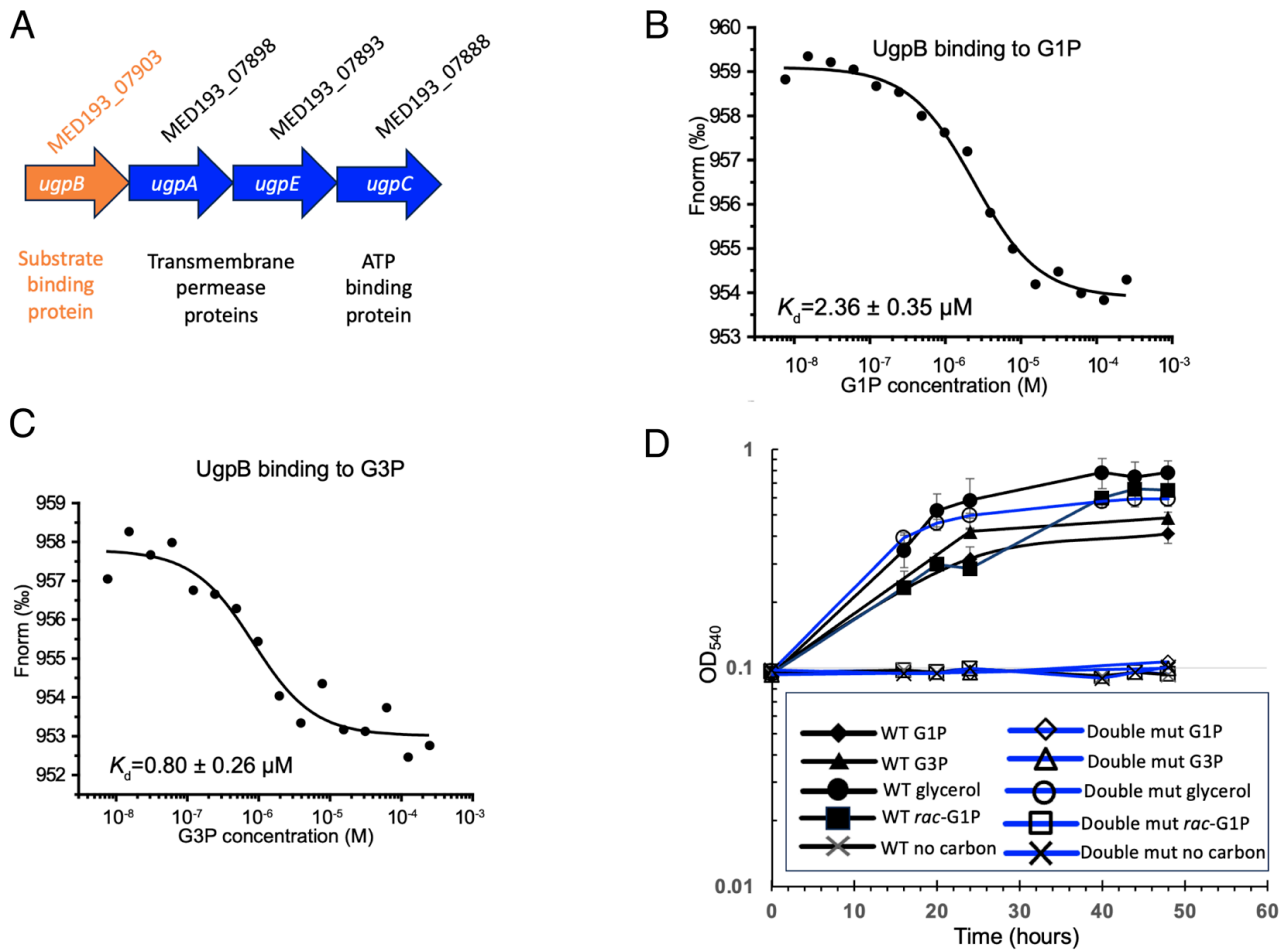
UgpB is the second G1P binding protein in *Phaeobacter* sp. MED193. (*A*) Gene neighborhood of the *Phaeobacter* sp. MED193 UgpB transporter, including the substrate-binding protein UgpB, the ATP binding protein UgpC and the transmembrane proteins UgpA and UgpE. (*B*) *Phaeobacter* sp. MED193 UgpB binding to G1P. The change in MST signals was fitted (black lines) to yield a *K_d_* of 2.36 µM for G1P. (*C*) *Phaeobacter* sp. MED193 UgpB binding to G3P. The change in MST signals was fitted (black lines) to yield a *K_d_* of 0.80 µM for G3P. (*D*) Growth of the *gpxBugpB* double mutant (blue lines) and wild type (black lines) *Phaeobacter* sp. MED193 on 5 mM G1P (diamonds) and G3P (triangles) as the sole C source. Glycerol (circles) is used as a positive control and a no carbon negative control (crosses) is also included. Rac-G1P is a 1:1 racemic mixture of L-glycerol 3-phosphate (G3P) and L-glycerol-1-phosphate (G1P).

Given that GpxB binds both G1P and G3P, we next investigated whether the bacterial G3P transporter UgpB (Fig. 2*A*) is also capable of binding G1P although UgpB has thus far only been shown to transport G3P (21). To assess whether UgpB can bind G1P, we heterologously overexpressed the *Phaeobacter* UgpB homologue (MED193_07903) and performed binding assays (*SI Appendix*, Fig. S6). Among the substrates tested, recombinant UgpB had binding affinities (*K*_d_) of 2.36 ± 0.35 μM for G1P and 0.80 ± 0.26 μM for G3P, suggesting UgpB can transport both G1P and G3P in this bacterium (Fig. 2 *B* and *C*). GpxB and UgpB appear to be the sole substrate-binding proteins for G1P and G3P in this bacterium, as a ∆*ugpB*∆*gpxB* double mutant was unable to grow on either substrate as the sole carbon source, although growth on glycerol remained unaffected (Fig. 2*D*). These findings collectively indicate that UgpB and GpxB function as the primary transporters for G1P and G3P in this bacterium.

It is striking to note that both glycerol phosphate-binding proteins, UgpB and GpxB, show weak-to-no selectivity against G1P and G3P, with their *K*_d_ values for G1P and G3P differing by less than three-fold. This is in sharp contrast to the G1P and G3P biosynthesis enzymes, G1P dehydrogenase (E.C. 1.1.1.261), and G3P dehydrogenase (E.C. 1.1.1.94), which catalyze the stereospecific reduction of dihydroxyacetone phosphate to form G1P and G3P, respectively (22). Structural and biochemical studies have demonstrated that such chiral selectivity in these glycerol phosphate dehydrogenases arises from the stereochemical mechanism of hydride ion transfer between the substrate (dihydroxyacetone phosphate) and the nicotinamide ring of the reductant NADH. While G3P dehydrogenase transfers the hydride ion from the *si* face of the nicotinamide ring to the substrate, G1P dehydrogenase transfers the hydride ion from the *re* face (23–25).

### Elucidation of the Contrasting G1P Binding Sites in GpxB and UgpB.

Although both GpxB and UgpB can function as G1P transporters in this bacterium, they lack sequence similarity. Thus, questions arise as to how these proteins may have evolved to bind glycerol phosphates. Substrate-binding proteins are part of a structurally related class, which can be grouped into seven well-defined clades (26, 27). GpxB is classified under the phosphonate uptake transporter (PhnT) family (3.A.1.9) within the D-III clade, with a predicted molecular weight (MW) of 35.0 kDa. In contrast, UgpB belongs to the carbohydrate uptake (CUT1) transporter family (3.A.1.1) of the D-I clade and has a predicted MW of 47.2 kDa.

**Fig. 3.**
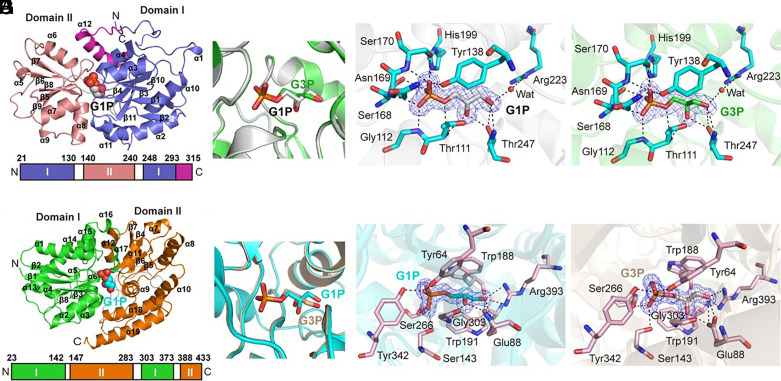
Structural comparison of GpxB^DSM11874^ and UgpB binding to G1P. (*A*) The overall structure of the GpxB^DSM11874^–G1P complex (*Top*). Domain I, domain II, and the C-terminal helix are colored in slate, salmon, and magenta, respectively. The G1P ligand in GpxB^DSM11874^ is shown as gray (carbon), red (oxygen), and orange (phosphorus) spheres. Block diagram representation (*Bottom*) of each domain in GpxB^DSM11874^ colored as in the top. The amino acids that determine the border of each domain are labeled as numbers. (*B*) Structural overlay of the binding pocket of the GpxB^DSM11874^–G1P complex and GpxB^DSM11874^–G3P complex. G3P is shown as green (carbon), red (oxygen), and orange (phosphorus). (*C*) A detailed view of the GpxB^DSM11874^–G1P binding sites. G1P is colored in gray and GpxB^DSM11874^ residues involved in binding G1P are colored in cyan. The water molecule is displayed as a red sphere. The *2F_o_* − *F_c_* densities for the G1P molecule are contoured in blue at 1.0σ. Dashed lines represent possible hydrogen bonds. (*D*) A detailed view of GpxB^DSM11874^ G3P binding sites. G3P is colored in green and GpxB^DSM11874^ residues involved in binding G3P are colored in cyan. The water molecule is displayed as a red sphere. The *2F_o_* − *F_c_* densities for G3P are contoured in blue at 1.0σ. Dashed lines represent possible hydrogen bonds. (*E*) The overall structure of the UgpB-G1P complex (*Top*) highlighting domain I (green) and domain II (orange) with the G1P molecule in UgpB. Block diagram representation (*Bottom*) of each domain in UgpB colored as in the top. The amino acids that determine the border of each domain are labeled as numbers. (*F*) Structural superposition of the binding pocket of the UgpB-G1P complex (cyan) and the UgpB-G3P complex (wheat) with the G1P and G3P ligands shown as sticks. (*G*) Residues involved in binding G1P in UgpB. The G1P molecule is shown as cyan sticks and UgpB residues as pink sticks. The *2F_o_* − *F_c_* densities for the G1P molecule are contoured in blue at 1.0σ. Possible hydrogen bonds are drawn as dashed lines. (*H*) Residues involved in binding G3P in UgpB. The G3P molecule is shown as wheat sticks and UgpB residues as pink sticks. The *2F_o_* − *F_c_* densities for G3P are contoured in blue at 1.0σ. Possible hydrogen bonds are drawn as dashed lines.

Since GpxB and UgpB are the only known transporters for G1P to date, to uncover the structural basis of G1P binding in these proteins, we solved both structures using GpxB from the gammaproteobacterium *Marinobacter* sp. DSM11874 (hereafter GpxB^DSM11874^) in complex with G1P and G3P. GpxB^DSM11874^ possesses 50% sequence identity to that of *Phaeobacter* sp. MED193 and also showed highest binding toward G1P (*K*_d_ 1.72 ± 0.28 µM) and G3P (*K*_d_ 1.06 ± 0.15 µM) among the C_3_ phosphates tested (*SI Appendix*, Fig. S2). Its structure was solved at a resolution of 1.68 Å and 2 Å in complex with G1P and G3P, respectively, with a rmsd of 0.09 Å (*SI Appendix*, Table S3). The overall structure of GpxB^DSM11874^ exhibits the typical topology of type II periplasmic binding proteins (PBPs) (28), with two domains separated by a hinge region containing two loops (residues 131–139 and 241–247) (Fig. 3*A*). G1P and G3P are aligned in the binding pockets in a similar manner (Fig. 3*B*) with the phosphate “head” group coordinated by Gly112, Thr111, Tyr138, Ser168, Asn169, Ser170, and His199, whereas the two hydroxy groups on the “tail” end of G1P and G3P are coordinated through hydrogen bonding with Thr111, and Thr247 (Fig. 3 *C* and *D*). Additionally, the side chain of Arg223 forms another hydrogen bond with the C3 hydroxyl group of G1P or C1 hydroxyl group of G3P, respectively, via a water molecule (Fig. 3 *C* and *D*). Site-directed mutagenesis experiments confirmed the crucial role of both phosphate-coordinating residues (e.g., Tyr138, His199) and hydroxy groups-coordinating residues (Thr247, Arg223) in G1P binding and the corresponding alanine mutants exhibited a 10- to 100-fold reduction in binding affinity (*SI Appendix*, Fig. S7).

The UgpB structure of *Phaeobacter* sp. MED193 was also solved in the presence of G1P and G3P ligands (*SI Appendix*, Table S3). The overall structure of UgpB contains two α/β domains connected via three hinges (residues 143−146, 284-302, and 374−387) and a G1P molecule occupies the active-site between the two domains. Domain I (residues 23−142 and 303−373) and domain II (residues 147−283 and 388−433) are composed of a four-stranded β-sheet surrounded by 11 α-helices and a four-stranded β-sheet surrounded by 8 α-helices, respectively (Fig. 3*E*). Again, both G1P and G3P occupy the same binding pockets in UgpB (Fig. 3*F*). The two hydroxyl groups of G1P/G3P are in close contact with Gly303, Arg393 and Glu88 by hydrogen bonds whereas the phosphate head group is coordinated by Tyr64, Ser143, Ser266, and Tyr342 (Fig. 3 *G* and *H*). A noticeable feature of the UgpB binding pocket, that makes it distinct from GpxB, is the presence of two highly conserved Trp residues (Trp188, Trp191) in UgpB which provides π-stacking interactions and forms a clamp holding the glycerol moiety of the linear G1P/G3P (Fig. 3 *G* and *H*) (21). Moreover, Trp191 also forms O-H···π hydrogen bonding with the oxygen atom of the C2 hydroxyl group of G3P (3.4 Å) (29) (Fig. 3*H*). Indeed, mutagenesis analyses confirmed the key role of all these Trp residues in securing G1P and G3P in its binding pocket (*SI Appendix*, Fig. S8). Thus, although both UgpB and GpxB can bind to G1P (and G3P), their binding pockets are strikingly different.

### Emergence of G1P-Binding Sites in the PhnT Family.

**Fig. 4.**
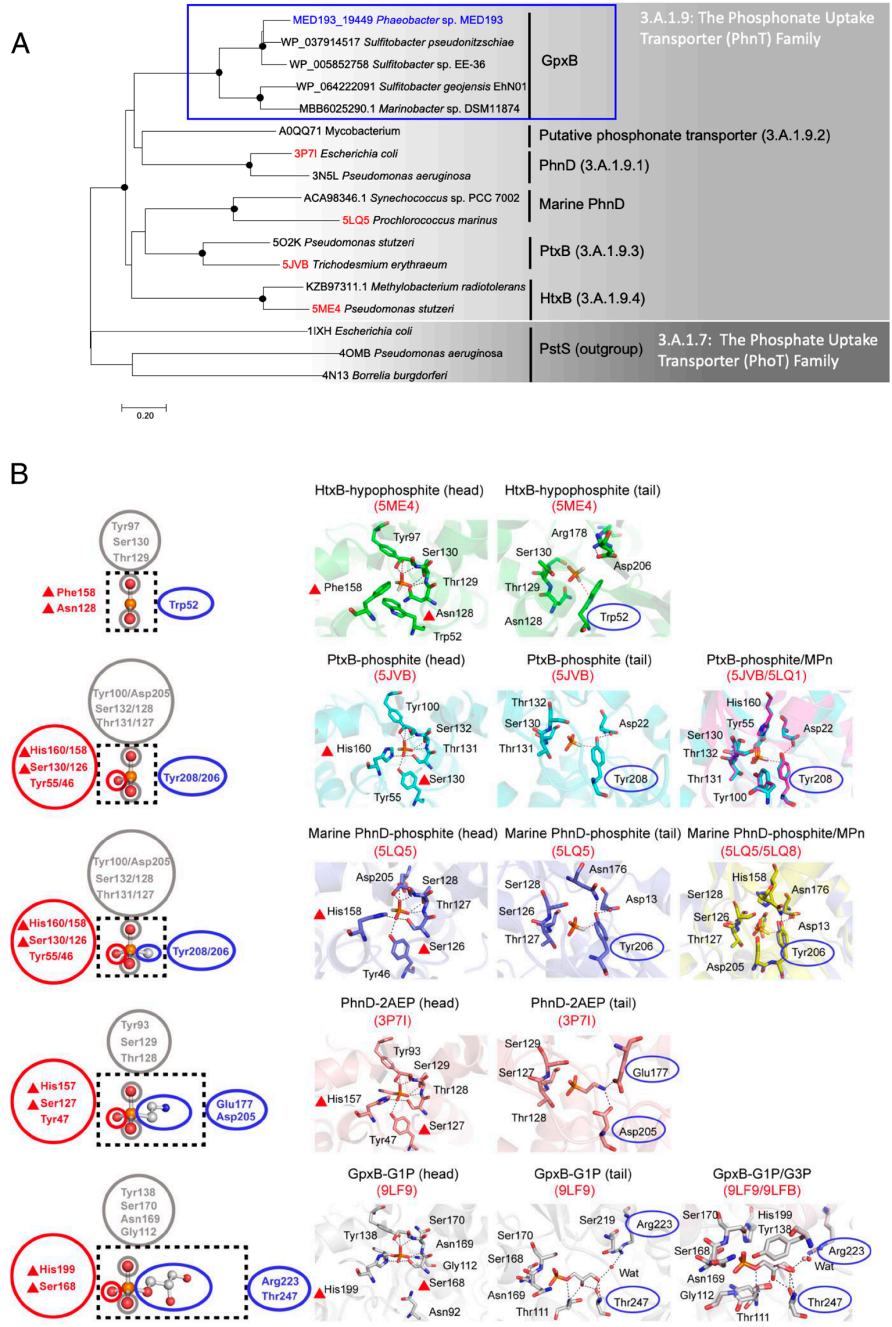
Evolution of glycerol phosphate binding in the PhnT family. (*A*) Phylogenetic tree showing the relationship between GpxB proteins of the PhnT family (3.A.1.9). In the Transporter Classification Database (https://www.tcdb.org/), four members of the PhnT family associated with organic phosphonate transport are known, including C-P lyase linked PhnD (3.A.1.9.1), an uncharacterized *Mycobacterium* phosphonate transporter (3.A.1.9.2), a phosphite transporter (PtxB, 3.A.1.9.3), hypophosphite transporter (HtxB, 3.A.1.9.4), as well as unclassified PhnD homologues in marine *Synechococcus* and *Prochlorococcus* (marine PhnD). PstS phosphate binding proteins belonging to the phosphate uptake transporter family (3.A.1.7) are used as the outgroup. (*B*) Schematic plot (*Left*) and detailed view (*Right*) of the evolution of phosphonate binding pockets and key residues coordinating the phosphorus-containing head and the non-phosphorus-containing tail. Key evolving residues involved in binding the head and tail of the phosphonate are highlighted with red triangles and blue ovals, respectively. As the head evolves from hypophosphite to phosphite groups, a conserved histidine and a conserved serine (red triangles) evolved to help stabilize the oxygen atom of the third P-O binding site. Meanwhile, an increase in volume of the binding pockets appears while the tail grows from a single H atom to more complex hydroxy groups in G1P and G3P (*SI Appendix*, Table S4). As the tail evolves from a single H atom to a -CH_3_ group, a conserved aromatic amino acid residue evolved for hydrogen bonding with the hydrogen atom in (hypo)phosphite or methylphosphonate through a P-H···π or a C-H···π bond (Trp52 in HtxB, Tyr208 in PtxB, Tyr206 in marine PhnD). Finally, a pair of Arg223/Thr247 residues in GpxB evolved to orient the two hydroxyl groups in G1P/G3P, while a pair of conserved Glu177/Asp205 residues in PhnD evolved to coordinate the positively charged -NH_3_^+^ group in 2AEP. In the schematic plot, the ligands are displayed as ball-and-stick and black dashed boxes represent the substrate-binding pockets. Residues in red and gray circles are involved in binding the phosphorus-containing head. In the detailed view of the ligand binding sites, proteins are shown as cartoons, with the bound ligands and selected residues shown as sticks. The water molecule is displayed as a red sphere. Black dashed lines represent possible hydrogen bonds and P-H…π and C-H…π interactions are drawn as red dashed lines.

Identification of these two contrasting G1P-binding proteins in two different families of transporters thus provides a unique opportunity to uncover the trajectory of the formation of two contrasting G1P binding sites from a phylogenetic and evolutionary perspective. Phylogenetic analyses placed GpxB on a distinct branch within the PhnT family (Fig. 4*A*), together with substrate-binding proteins involved in transporting hypophosphite (H_2_PO_2_H), phosphite (H_2_PO_3_H), and small phosphonates like methylphosphonate (CH_3_-PO_3_H_2_), 2’-aminoethylphosphonate (2AEP, NH_2_-CH_2_-CH_2_-PO_3_H_2_). While the P-containing head of these substrates remains similar in size and charge, the tail group varies significantly, expanding from single H atoms in (hypo)phosphite to larger, more complex aminoethyl- and dihydroxypropanone groups in phosphonates. Correspondingly, the binding pockets of these proteins have evolved to accommodate this increase in tail size. Indeed, GpxB, which binds to G1P/G3P, has the largest binding pocket, while HtxB, which binds hypophosphite, has the smallest (Fig. 4*B* and *SI Appendix*, Table S4). An evolutionary pathway for key residues involved in coordinating head and tail becomes evident through comparative structural analysis of PhnT family proteins. For example, while residues involved in binding the P-containing head are largely conserved, a new histidine and a serine emerged in PtxB, PhnD, and GpxB (red triangles, Fig. 4*B* and *SI Appendix*, Fig. S9*A*) to coordinate the third oxygen moiety as the head evolves from two P-O binding sites (hypophosphite) to three P-O binding sites (phosphite, G1P/G3P, and other phosphonates). Similarly, in HtxB and PtxB, which binds hypophosphite and phosphite, respectively (30), a crucial P-H···π hydrogen bond orients the hydrogen atom attached to P in (hypo)phosphite, with a conserved aromatic residue playing a key role (Trp52 in HtxB and Tyr208 in PtxB). This aromatic tyrosine cap appears to help stabilize the closed state of the protein and acts as a steric barrier for blocking the binding of ligands with larger R groups (30). However, in GpxB, such aromatic residues do not exist in corresponding positions (*SI Appendix*, Fig. S9*A*). As the tail of the substrate expands from a hydrogen atom to a positively charged amine (in 2AEP) or hydroxyl groups (in G1P and G3P), an increase in volume of the binding pockets appears along with the loss of the steric barrier tyrosine. Two key residues in PhnD, Glu177/Asp205, evolved to accommodate the -NH_2_ group (31), whereas in GpxB, these residues have been replaced by Arg223/Thr247, which form hydrogen bonds with the hydroxy groups in G1P and G3P (Fig. 4*B* and *SI Appendix*, Fig. S9*A*). This suggests that GpxB likely evolved from smaller (hypo)phosphite-binding proteins, with gradual changes in key residues to accommodate the increasing size, charge, and polarity of the tail group, ultimately enabling specific binding to G1P and G3P.

### Emergence of G1P-Binding Sites in the CUT1 Family.

**Fig. 5.**
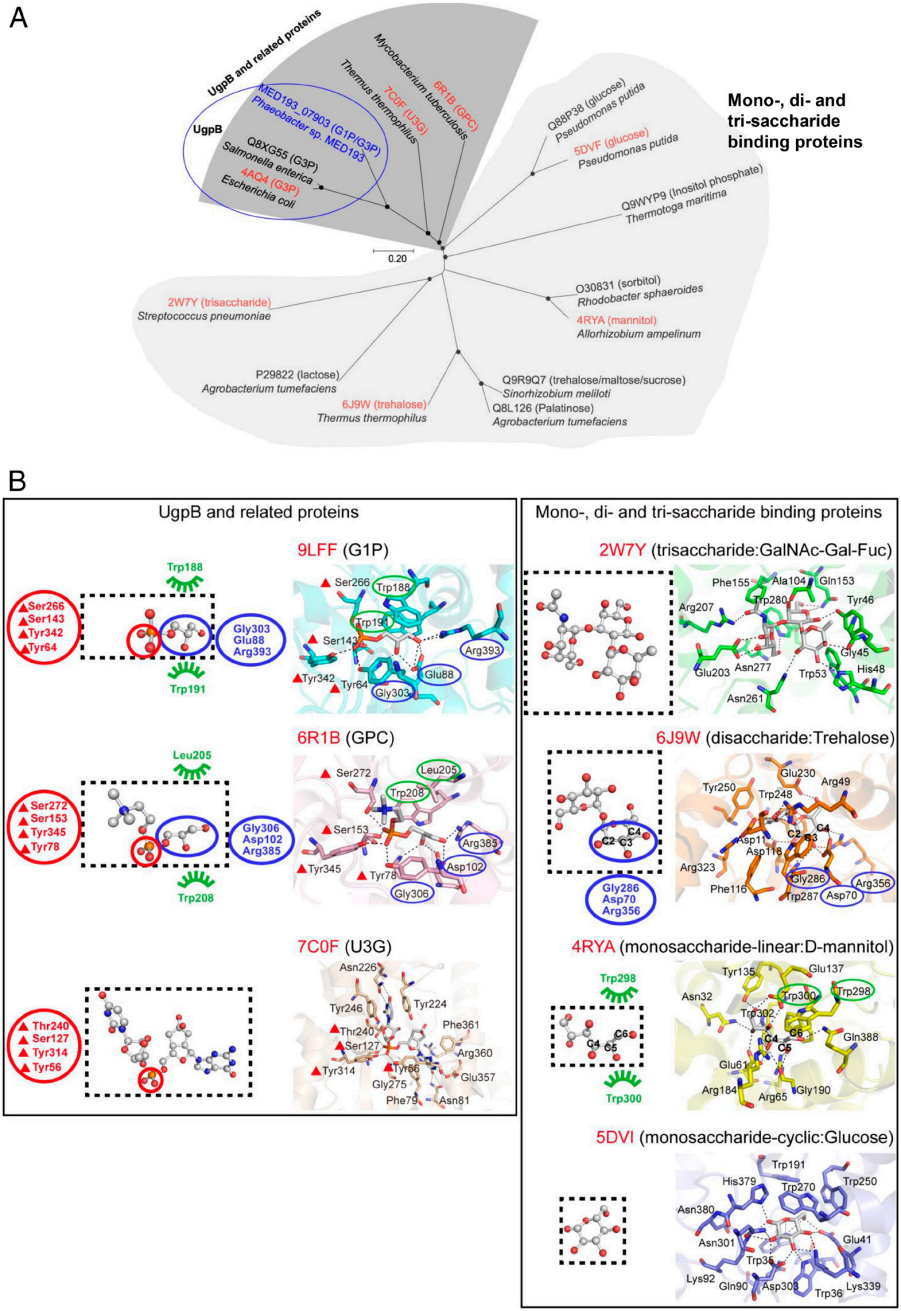
Evolution of glycerol phosphate binding in the CUT1 family. (*A*) A phylogenetic tree illustrating the relationships between UgpB (highlighted in a blue oval) and closely related members (highlighted in shaded gray) of the Carbohydrate Uptake Transporter-1 (CUT1) Family (3.A.1.1). (*B*, *Left*) A close examination of UgpB and its related members in the CUT1 family reveals connections to a GPC binding protein from *M. tuberculosis* (33) and a U3G binding protein from *Thermus thermophilus* (32). There is a gradual increase in the size of the binding pockets from G1P/G3P binding proteins to GPC and U3G binding proteins (*SI Appendix*, Table S5). The evolution of key residues from the phosphate-containing U3G to GPC to G1P/G3P is also illustrated, highlighting two conserved Tyr residues (64/342) and two Ser residues (143/266) involved in orienting the oxygen atoms of P-O in phosphate (red triangles). In addition, two conserved Trp residues (188/191) emerged in binding the glycerol moiety of the linear G1P/G3P in UgpB (green ovals). (*Right*) The evolution of binding pockets depicted by surface representation of proteins demonstrating a transition from trisaccharides to disaccharides to monohexose, along with a decrease in the volume of the substrate-binding pockets (*SI Appendix*, Table S5). Noticeably, the two hydroxyl groups in G1P/G3P are akin to the hydroxy groups of C2-C4 of the first glycosyl unit of trehalose as well as those in D-mannitol. A comparison of the binding pockets of trehalose-binding protein and G1P/G3P binding UgpB shows that three residues (Gly286, Asp70, Arg356, and Gly303, Glu88, Arg393 respectively) involved in coordinating the oxygen atoms of the glycerol moiety are highly conserved (blue ovals). Similarly, the binding pocket of UgpB also contains two conserved Trp residues (188/191 in UgpB and 298/300 in the D-mannitol-binding protein 4RYA) involved in binding the glycerol moiety of the linear G1P/G3P (green ovals). In the schematic plot, ligands are displayed as ball-and-stick and black dashed boxes represent the substrate-binding pockets. Residues in red and gray circles are involved in binding the phosphorus-containing head. In the detailed view of the ligand binding sites, proteins are shown as cartoons with the bound ligands and selected residues shown as sticks. Key evolving residues involved in binding the phosphate group and glycerol moiety are highlighted with triangles and ovals. Possible hydrogen bonds are drawn as dashed lines.

The CUT1 family of transporters can bind a variety of tri-, di-, and monosaccharides, as well as phosphorylated glycerols and their derivatives. Unsurprisingly, the evolution of the CUT1 family appears more complex. One notable evolutionary pathway for some members involves the progressive shrinking of binding pockets, transitioning from trisaccharides to disaccharides and then to monosaccharides, as shown in Fig. 5*A* and *SI Appendix*, Table S5. The formation of monohexose-binding pockets likely marks a key branching point in the evolution of the CUT1 family, given the existence of both open-chain and cyclic forms of monohexose. A comparison of the ligands of these proteins shows some similarities between the glycerol moiety (two adjacent hydroxyl groups) of G1P/G3P and the hydroxy groups of the first sugar rings in trehalose and those of D-mannitol (Fig. 5*A*). There are two likely evolutionary scenarios from sugar-binding proteins to glycerophosphate-binding proteins. In the first scenario, UgpB may evolve from oligosaccharide-binding proteins by retaining residues engaged in hydrogen bonding from the hydroxyl oxygen (Fig. 5*B*). Thus, in agreement with previous speculation (32), key residues involved in orienting the two hydroxy groups in UgpB (Gly303, Glu88 and Arg393) are conserved in the trehalose-binding protein (Gly286, Asp70 and Arg356). In support of this hypothesis, UgpB and the hexitol-binding protein (D-mannitol binding protein 4RYA) both contain two conserved Trp residues (188/191 in UgpB and 298/300 in the hexitol-binding protein) involved in forming a clamp to hold the glycerol moiety of the linear substrate G1P/G3P and D-mannitol (Fig. 5*B*). In the second scenario, UgpB may evolve from an open-chain monohexose-binding protein by incorporating phosphate as a “new” functional group in the substrate. Phylogenetically, UgpB, along with several other members of the CUT1 family, forms a distinct branch separate from proteins that bind disaccharides and oligosaccharides (Fig. 5*B*). A closer examination of the multiple sequence alignment reveals two proteins closely related to UgpB: one from *Mycobacterium tuberculosis*, the glycerophosphocholine (GPC)-binding protein (33), and the other the uridylyl-3’-5’-phosphoguanosine (U3G)-binding protein (32), both of which contain a phosphate group (Fig. 5*B*). Remarkably, two Tyr residues and two Ser residues that coordinate the phosphate P-O bond (red triangles) are strictly conserved in UgpB, as well as in the GPC and U3G-binding proteins (*SI Appendix*, Fig. S9*B*), but not in glucose-binding proteins. Moreover, the residues forming hydrogen bonds with the hydroxyl oxygen of the glycerol moiety still exist in GPC-binding proteins (Gly306, Asp102 and Arg385) (Fig. 5*B*). During the evolution from GPC and U3G-binding proteins to UgpB, the volume of their binding pockets shrinks to accommodate the decrease in ligand size (*SI Appendix*, Table S5), and at the same time, two conserved Trp residues (188/191) appear to form a clamp to hold the glycerol moiety of the linear G1P/G3P (Fig. 5*B*). This evolution results in the reshaping of the binding pocket to accommodate the negatively charged phosphate moiety and supports the hypothesis that UgpB may have evolved from a non-phosphate-containing linear hexose-binding protein.

Interestingly, structural comparison of the GpxB and UgpB proteins also provides insights into the evolutionary trajectories of the PhnT and CUT1 protein families. Proteins in the PhnT family appear to evolve from a phosphorus-containing head toward a non-phosphorus-containing tail, whereas the CUT1 family exhibits the opposite trend, evolving from non-phosphorus-containing structures toward phosphorus-containing heads. In the binding cavity of GpxB, five water molecules are located near the tail of G1P (*SI Appendix*, Fig. S10*A*). Similarly, five water molecules were observed behind the -NH_2_ group of 2AEP in the binding cavity of *Ec*PhnD (31). These water molecules may create a spatial foundation for binding phosphonates/organophosphorus compounds with longer R1 groups during the evolution of the PhnT family (*SI Appendix*, Fig. S10*A*). Conversely, in UgpB, five water molecules occupy the head region of the phosphorus-containing G1P within the binding cavity, potentially supporting an evolutionary transition from a glycerol sidechain to a phosphorus-containing head, and ultimately to the choline moiety of GPC (*SI Appendix*, Fig. S10*B*). The binding affinity of GpxB and UgpB is further reflected in their hydrogen bond networks. GpxB exhibits a more extensive network, with 13 hydrogen bonds stabilizing the binding of G1P, including seven hydrogen bonds specifically with the phosphate head group (Fig. 3*C*). In contrast, UgpB forms 10 hydrogen bonds in total, with only four engaging the phosphate head group (Fig. 3*G*). The denser hydrogen bond network in GpxB suggests it likely binds the phosphate group more strongly than UgpB. Additionally, residues in UgpB’s binding pocket play a particularly significant role at the individual residue level, as demonstrated by site-directed mutagenesis experiments (*SI Appendix*, Figs. S7 and S8). This highlights the structural importance of these residues for UgpB’s binding function. Thus, while both GpxB and UgpB can bind to G1P (and G3P), they exhibit striking structural and functional differences that reflect their distinct evolutionary and biochemical characteristics.

## Conclusions

In summary, we have identified two G1P transporters and elucidated the molecular mechanisms underlying their high-affinity binding to G1P. Our findings highlight a remarkable instance of functional convergent evolution, showcasing how two seemingly unrelated proteins have adapted to bind the same substrate to support microbial growth. High-affinity transporters for G1P are crucial for its uptake, and further investigation into the regulatory mechanisms governing GpxB and UgpB will provide deeper insights into the roles of these transporters in G1P metabolism. Given the vast abundance of Archaea in the global oceans, G1P cycling is likely to be particularly pronounced in these waters. The prevalence of G1P transporter proteins in the global ocean microbiome suggests that G1P cycling plays a significant yet often overlooked role in the global C and P cycles.

## Materials and Methods

### Strains and Culture Conditions.

*Phaeobacter* sp. MED193 and its mutant strains were routinely maintained on 1.5% (w/v) Difco marine broth 2216 agar with antibiotics as appropriate. To test the growth of *Phaeobacter* sp. MED193 on G1P/G3P, cells were first cultivated in 5 mL marine broth medium overnight at 30 °C with shaking at 150 rpm. A 1% (v/v) inoculum was then transferred to defined artificial seawater medium supplemented with 50 µM phosphate (6). After 24 h growth, 12.5 mL cultures were collected by centrifugation and inoculated into 100 mL artificial seawater medium. Subsequently, either G1P or G3P (purchased from Sigma-Aldrich, St. Louis, USA) was added to a final concentration of 5 mM as the sole C source. Cells (40 mL) were harvested after 48 h and used for proteomics.

All *Escherichia coli* strains were routinely cultivated in LB medium supplemented with antibiotics as appropriate. To express G1P/G3P substrate-binding proteins, genes were chemically synthesized (see below) and subcloned into pET-22b or pET-28a vectors and transformed into *E. coli* BL21 (DE3). *E. coli* cells were routinely cultivated in LB medium at 37 °C until the OD_540_ reached 0.3 to 0.5 before protein expression was induced by adding 0.1 to 0.5 mM isopropyl β-D-1-thiogalactopyranoside (IPTG) (final concentration) and cultures shifting to 15 to 22 °C for a further 6 to 12 h before harvesting by centrifugation.

### Proteomics Analysis.

For cellular proteomes, cell pellets were resuspended in 1 mL ddH_2_O and 200 µL LDS buffer was added. For exoproteomes, proteins were precipitated from cell-free culture medium using trichloroacetic acid as described previously (34). Cellular and exo protein samples were then boiled at 98 °C for 15 min before loading onto a precast NuPAGE Bis-Tris gel for a short run at 140 V for 5 to 15 min. After staining with Coomassie blue and destaining overnight in ddH_2_O, protein bands were cut into small pieces and extracted as described previously (34). Trypsin digested peptides were cleaned using Costar Spin-X centrifuge tube filters and analyzed by nanoLC (Ultimate 3000)-ESI MS/MS (Orbitrap Fusion mass spectrometer, ThermoFisher) at the University of Warwick Research Technology Platform. The recorded MS/MS data was processed using MaxQuant (v1.5.5.1) and Perseus (v1.6.5.0). Statistical analysis was set using a false discovery rate of 0.05 and a minimal log_2_ fold change of two. Only proteins that were present in every biological replicate of at least one condition (G1P, G3P, or glycerol) were retained for statistical analyses.

### Microscale Thermophoresis.

The binding affinity of the purified proteins to G1P and other structurally related chemicals was measured using a Monolith NT.115 (NanoTemper Technologies, Munich, Germany). Proteins (160 nM) were labeled in assay buffer (PBS containing 0.05% (v/v) Tween-20) using the Protein Labelling Kit RED-Tris-NTA (NanoTemper Technologies). For binding assays, the buffer was replaced by 20 mM HEPES pH 7.4, 100 mM NaCl, 0.05% (v/v) Tween-20. Each chemical was prepared by serial dilution in the same assay buffer and mixed with the same volume of labeled protein (at a final concentration of 80 nM). The mixture was then loaded onto Monolith™ NT.115 Series capillaries (NanoTemper Technologies, Germany) and thermophoresis was measured at 22 °C using 60% excitation power and medium MST power. Data were analyzed using MO. Affinity Analysis v2.3 software (NanoTemper Technologies).

### Construction of *Phaeobacter* Mutants.

The *gpxBugpB* double mutant was generated using a conjugation protocol as described previously (35). First, two homologous regions of an upstream and a downstream fragment flanking the *gpxB* gene were cloned into suicide plasmid pK18*mobsacB*, together with a spectinomycin (Spec) resistance cassette which was inserted in between the upstream and downstream fragments. The resulting plasmid was then mobilized into *Phaeobacter* sp. MED193 using *E. coli* S17.1 lambda *pir* and transconjugants selected on marine broth MB agar containing Spec (200 µg mL^−1^). Putative mutants were confirmed by replicate plating on MB agar with Spec and Kan respectively and those colonies that did not grow on Kan plates were further validated by PCR using primers targeting the gene of interest. The same protocol was repeated to generate the *gpxBugpB* double mutant in the *gpxB* single mutant background, except that a Gentamycin (Gm) resistant cassette was inserted in between the upstream and downstream fragments of *ugpB* into pK18*mobsacB*. All primers, plasmids, and bacterial strains used are listed in *SI Appendix*, Table S6.

### Gene Synthesis, Point Mutations, and Protein Overexpression and Purification.

The full-length codon optimized GpxB (MED193_19449) of *Phaeobacter* sp. MED193 (WP_009811375.1), and four GpxB homologs (*Marinobacter* sp. DSM11874, MBB6025290.1; *S. pseudonitzschiae* DSM26824, WP_037914517.1; *Sulfitobacter* sp. EE-36, WP_005852758.1; *S. geojensis* sp. EhN01, WP_064222091.1) were chemically synthesized by Beijing Tsingke Biotech Co., Ltd. (China), and subcloned into pET-22b (Novagen, America) with a C-terminal His-tag. An N-terminal signal peptide truncated [residues 1−22 predicted by SignalP 5.0 Server (36)] MED193_07903 gene encoding UgpB from *Phaeobacter* sp. MED193 (WP_009809101.1) was synthesized by Beijing Tsingke Biotech Co., Ltd. (China), and then subcloned into pET-28a (Novagen, America) providing an N-terminal His-tag. All point mutations of GpxB^DSM11874^ and UgpB were generated via a PCR-based method using PfuUltra II Fusion HS DNA Polymerase (Agilent Technologies, America) and verified by DNA sequencing. All proteins were expressed in *E. coli* BL21 (DE3). *E. coli* cells were routinely cultivated in LB medium supplemented with 100 μg mL^−1^ ampicillin (30 μg mL^−1^ kanamycin for UgpB and its mutants) at 37 °C until the OD_600_ reached 0.8 to 1.0 before protein expression was induced by adding 0.5 mM IPTG and cultures shifting to 17 °C for a further 14 to 16 h before harvesting by centrifugation. Proteins were first purified by affinity chromatography with Ni^2+^-nitrilotriacetic acid (NTA) resin (Qiagen, Germany) and then fractionated by a Superdex 200 column (Cytiva, America) with buffer containing 10 mM Tris-HCl (pH 8.0), 100 mM NaCl.

### Crystallization and Data Collection.

The purified proteins used for crystallization were prepared to 7 mg mL^−1^ (GpxB^DSM11874^) and 8 mg mL^−1^ (UgpB) in 10 mM Tris-HCl (pH 8.0), 100 mM NaCl. To obtain the structure of the GpxB^DSM11874^–G1P complex, GpxB^DSM11874^ was cocrystallized with 2 mM G1P using the sitting-drop vapor diffusion method at 18 °C for 2 wk using a protein:reservoir solution ratio of 1:1. Crystals of the GpxB^DSM11874^–G1P complex were obtained in sitting drops containing 0.04 M potassium phosphate, 16% (w/v) polyethylene glycol (PEG) 8000 and 20% (v/v) glycerol. Crystals of the UgpB-G1P complex were obtained using the same cocrystallization method, but in 0.1 M PCB buffer (the molar ratios of sodium propionate to sodium cacodylate to BIS-TRIS propane were 2:1:2) (pH 5.0), 25% (w/v) PEG 1500. Crystals of the UgpB-G3P complex were obtained using the same cocrystallization method, but in conditions containing 0.1 M MIB buffer (the molar ratios of sodium malonate to imidazole to boric acid were 2:3:3) (pH 7.0), 25% (w/v) PEG 1500 in the presence of 2 mM G3P. For the GpxB^DSM11874^–G3P complex, GpxB^DSM11874^ was cocrystallized with 2 mM G3P using a hanging-drop vapor diffusion method in crystallization buffer containing 0.1 M calcium acetate, 0.1 M sodium acetate (pH 4.5), 10% (w/v) PEG 4000 at 18 °C for 2 wk.

The X-ray diffraction data of GpxB^DSM11874^–G3P complex was collected by X-ray single crystal diffractometry at the State Key Laboratory of Microbial Technology of Shandong University and initial diffraction datasets were processed using the HKL3000 program with default settings (37). All other X-ray diffraction data were collected on the BL18U1 and BL19U1 beamlines at the Shanghai Synchrotron Radiation Facility.

### Structure Determination and Refinement.

Crystals of the GpxB^DSM11874^–G1P/G3P complex and the UgpB-G1P/G3P complex belonged to the *I*23 and *P*2_1_2_1_2 space group. The crystal structure of the GpxB^DSM11874^–G1P complex was determined by molecular replacement using a CCP4 program Phaser (38) with the structure of GpxB^DSM11874^ generated by AlphaFold2 (39) as the search model. The structure of the GpxB^DSM11874^–G3P complex was further determined using the same method, but with the solved GpxB^DSM11874^–G1P structure as a model. The crystal structures of the UgpB-G1P complex and UgpB-G3P complex were solved using the same method. Several rounds of refinement of all structures were carried out in Coot (40) and *Phenix* (41) using default parameters. All structure figures were processed using the PyMOL program (http://www.pymol.org/). The pocket volume of proteins was calculated using PyVOL 1.7.6 (42).

### Circular-Dichroism (CD) Spectroscopic Assays.

GpxB (and its mutants) and UgpB (and its mutants) were prepared to ~0.4 mg mL^−1^ in 10 mM Tris-HCl (pH 8.0), 100 mM NaCl. CD spectroscopic assays for these proteins were conducted on a J-1500 Spectrometer (Jasco, Japan) at 25 °C. All the data were collected under the following conditions: wavelength from 250 to 200 nm, scanning speed 500 nm min^−1^, bandwidth 1 nm, data pitch 0.5 nm and data interval 0.5 nm.

### GpxB and UgpB in Bacterial Genomes and the Global Ocean Metagenomes and Metatranscriptomes.

*Phaeobacter* sp. MED193 GpxB was used to search for its homologues in bacterial and archaeal genomes in the NCBI nonredundant protein sequences database using 1e^−50^ and sequence coverage >75% as the cut-off value. Tara Oceans metagenomes (OM-RGCv2+G) and metatranscriptomes (OM-RGCv2+T) were queried for GpxB and UgpB via the Ocean Gene Atlas (OGA) web portal as described previously (6). GpxB and UgpB abundance in both metagenomes and metatranscriptomes was obtained by using hmmsearch with an expected threshold of 1e^−80^ normalized to the median abundance of 10 single-copy marker genes as described previously (43).

## Supplementary Material

Appendix 01 (PDF)

## Data Availability

All structural data generated in this study has been deposited in the Protein Data Bank (PDB) database (www.pdb.org) with accession code 9LF9 (44), 9LFB (45), 9LFF (46), and 9LFJ (47) for GpxB^DSM11874^–G1P, GpxB^DSM11874^–G3P, UgpB-G1P, and UgpB-G3P, respectively.
