# Supplementary material for: Structural basis and evolutionary pathways of glycerol-1-phosphate transport in marine bacteria
Source: Proc Natl Acad Sci U S A. 2025 Dec 9;122(50):e2524546122. doi: 10.1073/pnas.2524546122 (PMC12718374; doi:10.1073/pnas.2524546122)
Supplement: Supplementary file 1 — Appendix 01 (PDF) [file pnas.2524546122.sapp.pdf]

Supplementary Information

Supplementary Tables

Table S1. Proteins differentially regulated in the cellular proteomes of *Phaeobacter* sp. MED193 when grown on G1P or G3P as the sole carbon source, compared to grown on glycerol.

Table S2. Distribution of the GpxB transporter in bacterial and archaeal genomes.

Table S3. Crystallographic data collection and refinement of GpxB and UgpB

Table S4. Volume of the protein binding pockets in the PhnT family.

Table S5. Volume of the protein binding pockets in the CUT1 family.

Table S6. List of oligonucleotides, plasmids and bacterial strains used in this study.

Supplementary Figure legends

Figure S1 Volcano plots of cellular proteomic data comparing *Phaeobacter* sp. MED193 grown on G3P as sole C source to MED193 cultured with glycerol.

Figure S2 Microscale thermophoresis (MST) binding affinity assay of purified *Phaeobacter* sp. MED193 GpxB (MED193_19449) and $GpxB^{DSM11874}$ (GpxB homolog from *Marinobacter* sp. DSM 11874) on a variety of substrates.

Figure S3 Microscale thermophoresis (MST) binding affinity assays of purified GpxB from several marine bacteria.

Figure S4 The occurrence of GpxB in bacterial and archaeal genomes.

Figure S5 The global distribution and expression of GpxB in the Tara Ocean microbiome.

Figure S6 Microscale thermophoresis (MST) binding affinity assays of purified *Phaeobacter* sp. MED193 UgpB (MED193_07903) on various substrates.

Figure S7 Characterisation of site-directed mutants of *Phaeobacter* sp. MED193 GpxB.

Figure S8 Characterisation of the site-directed mutants of *Phaeobacter* sp. MED193 UgpB.

Figure S9 Multiple sequence alignment of PhnT family (including GpxB) and CUT1 family proteins (including UgpB).

Figure S10 A comparison of the binding site cavity in GpxB and UgpB.

Table S1 Proteins differentially regulated in the cellular proteomes of *Phaeobacter* sp. MED193 when grown on G1P or G3P as the sole carbon source, compared to growth on glycerol. This Table includes corresponding locus tags, accession numbers and UniProt database annotations. The G1P/G3P transporter proteins are highlighted in red.

		G1P compared to glycerol				G3P compared to glycerol				
Annotation uniprot	Locus tag	Sign?	-LOG (*P*-val)	Rel. Abun (%)	log2 (FC)	Sign?	-LOG (*P*-val)	Rel. Abun (%)	log2 (FC)	Rel. Abun. in glycerol (%)
Phosphonate-binding periplasmic protein	MED193_19449	++	3.62	0.93	9.02	+	2.98	0.43	7.73	0.00
Phosphonates import ATP-binding protein	MED193_19454	+	2.45	0.36	7.13	+	2.28	0.19	6.26	0.00
Phosphonates ABC transporter permease protein	MED193_19459	+	3.30	0.15	4.65	+	2.96	0.13	4.36	0.01
Uncharacterized protein	MED193_19469	++	4.55	0.09	3.84	+	4.43	0.08	3.68	0.01
ABC transporter, ATP-binding/permease protein	MED193_05251	+	3.75	0.02	2.27		0.86	0.01	0.33	0.00
Amino acid permease	MED193_07459		1.24	0.02	2.10	+	2.75	0.03	2.70	0.00
Histidine kinase	MED193_19439	+	3.97	0.02	1.92	+	4.67	0.04	2.74	0.01
Outer membrane protein	MED193_08303	+	4.50	0.43	1.57	+	3.62	0.29	1.00	0.15
Phosphogluconate dehydratase	MED193_17424	+	4.29	0.22	1.53		0.33	0.08	0.05	0.08
YjeF family protein	MED193_05031		0.92	0.01	1.43	+	4.55	0.03	3.40	0.00
Glutathione-disulfide reductase	MED193_20839	+	4.57	0.39	1.26	+	4.28	0.27	0.76	0.17
Tol-Pal system protein TolB	MED193_09155	+	4.82	0.21	1.26	+	4.45	0.18	1.04	0.09
Universal stress family protein	MED193_17049	+	3.55	0.01	1.11	+	3.38	0.01	1.13	0.00
Peptidoglycan-binding protein	MED193_01380		3.13	0.21	1.08	+	4.52	0.22	1.18	0.10
Uncharacterized protein	MED193_03105	+	3.40	3.23	1.01		2.86	2.60	0.73	1.67
Hydrolase, NUDIX family protein	MED193_19764		0.09	0.01	0.25	+	3.56	0.02	1.58	0.01
Enoyl-CoA hydratase	MED193_03982		0.58	0.06	0.13	+	3.48	0.09	0.88	0.05
Sugar ABC transporter, sugar-binding protein	MED193_09570		0.31	0.68	-0.36	+	2.72	0.20	-2.04	0.89
Sugar ABC transporter, ATP-binding protein	MED193_09580		1.10	0.05	-0.61	+	3.95	0.01	-3.17	0.08
Sugar ABC transporter, ATP-binding protein	MED193_09575		1.65	0.08	-0.66	+	4.11	0.02	-2.83	0.14
Sugar ABC transporter, permease protein	MED193_09590		0.58	0.09	-0.78	+	4.03	0.01	-3.96	0.13
Uncharacterized protein	MED193_14802	+	3.82	0.12	-0.81		2.68	0.15	-0.41	0.22
Pyridine nucleotide-disulphide oxidoreductase	MED193_05534		2.10	0.01	-1.06	+	2.83	0.01	-1.51	0.02

Polyamine ABC transporter, permease protein	MED193_13448	+	3.26	0.03	-1.07		0.44	0.06	-0.13	0.07
Sugar ABC transporter, ATP-binding protein	MED193_10388	+	3.56	0.01	-1.09		0.04	0.03	0.01	0.03
Translation initiation factor IF-3	MED193_06664		0.77	0.02	-1.23	+	3.02	0.00	-3.10	0.04
Uncharacterized protein	MED193_07678	+	3.42	0.16	-1.46		2.19	0.73	0.78	0.45
Amino acid ABC transporter, substrate protein	MED193_05604	+	3.39	0.08	-2.46	+	4.64	0.04	-3.46	0.47
Transcriptional regulator	MED193_21244	+	3.64	0.01	-2.57		0.35	0.03	-0.16	0.03
Amino acid ABC transporter, ATP-binding protein	MED193_05624	+	3.47	0.00	-3.02	+	3.91	0.01	-2.73	0.04
Putative atp-binding abc transporter protein	MED193_07833	+	3.54	0.01	-3.27	+	3.13	0.01	-3.17	0.06
Uncharacterized protein	MED193_08513	+	3.18	0.00	-3.31		2.21	0.01	-2.52	0.05
Outer membrane protein	MED193_22031	+	3.56	0.00	-4.01		0.03	0.07	-0.03	0.07
Amino acid ABC transporter, ATP-binding protein	MED193_05619		1.88	0.01	-4.07	+	3.36	0.01	-3.61	0.08
Chloroacetaldehyde dehydrogenase	MED193_00725	++	3.91	0.02	-4.37	+	3.31	0.04	-3.10	0.32
Probable binding protein of sugar transporter	MED193_07818		2.29	0.01	-5.57	+	4.28	0.00	-5.47	0.22
Cobaltochelatase	MED193_19604	++	4.86	0.00	-7.19	++	4.49	0.00	-7.06	0.70

Sig?, Significance level at *P value < 0.05 (+) or < 0.01 (++).

Rel. Abun., Relative abundance of the protein in the cellular proteome.

Log2(FC), log2(fold change), normalized against glycerol grown cultures.

Table S2. Distribution of the GpxB transporter in bacterial and archaeal genomes.

Taxonomy	Number of Organisms
**. Bacteria**	**2600**
.. Pseudomonadota	2268
... Alphaproteobacteria	1150
.... Rhodobacterales	519
..... Roseobacteraceae	307
..... Paracoccaceae	202
..... unclassified Rhodobacterales	9
.... unclassified Alphaproteobacteria	9
.... Hyphomicrobiales	418
..... Rhizobiaceae	93
..... Devosiaceae	13
..... Stappiaceae	46
..... Cohaesibacteraceae	6
..... Notoacmeibacter ruber	1
..... Phyllobacteriaceae	30
..... Amorphaceae	5
..... Afifellaceae	2
..... Ahrensiaceae	4
..... Aurantimonadaceae	15
..... unclassified Hyphomicrobiales	10
..... Boseaceae	40
..... Methylobacteriaceae	55
..... Xanthobacteraceae	17
..... Hyphomicrobiaceae	12
..... Beijerinckiaceae bacterium	1
..... Pseudorhodoplanes sp.	1
..... Nitrobacteraceae	8
..... Chelatococcaceae	5
..... Phreatobacter sp.	1
..... Rhodoblastus sp.	1
..... Salinarimonadaceae	7
..... uncultured Alsobacter sp.	1
..... Rhabdaerophilum	3
..... Brucellaceae	30
..... Rhodobiaceae bacterium	1
..... Tepidamorphaceae bacterium	1
..... Methylocystis sp.	1
..... Kaistiaceae	2
..... Methylobrevis pamukkalensis	1
..... Anderseniella sp.	1
..... Propylenella sp.	1
..... Limoniibacter endophyticus	1
..... Prosthecodimorpha staleyi	1
.... Rhodospirillales	178
..... Kiloniellales bacterium	1
..... Rhodovibrionaceae	20

..... Rhodospirillaceae	8
..... Dongiaceae	2
..... Caenispirillum	2
..... Thalassospiraceae	44
..... Rhodospirillales bacterium	1
..... Magnetovibrio sp.	1
..... Azospirillum	2
..... Acetobacteraceae	64
..... Elioraea	6
..... Stellaceae	5
..... Kiloniellaceae	7
..... Thalassobaculaceae	12
..... Oceanibaculum	3
.... Sneathiellales	13
..... Sneathiellaceae	12
..... Sneathiellales bacterium	1
.... Geminicoccaceae	5
..... Geminicoccaceae bacterium	1
..... Geminicoccus	4
.... Sphingomonadales	2
..... Novosphingobium sp.	1
..... Sphingomonadales bacterium	1
.... Candidatus Pelagibacterales	3
..... Candidatus Pelagibacteraceae	2
..... Candidatus Pelagibacterales bacterium	1
.... Minwuiales bacterium	1
.... Acetobacterales bacterium	1
... Pseudomonadota bacterium	1
... Gammaproteobacteria	810
.... Oceanospirillales	381
..... Halomonadaceae	302
..... Hahellaceae	2
..... Saccharospirillaceae	10
..... Natronospirillum operosum	1
..... Oceanospirillaceae	62
..... Litorivicinaceae	2
..... Aestuariirhabdus haliotis	1
..... Oceanospirillales bacterium	1
.... Pseudomonadales	93
..... Marinobacteraceae	72
..... Pseudomonadales bacterium	1
..... Pseudohongiellaceae	7
..... Pseudomonadaceae	13
.... unclassified Gammaproteobacteria	5
..... Gammaproteobacteria bacterium	1
..... marine bacterium B5-7	1
..... Gammaproteobacteria bacterium 2W06	1
..... Gammaproteobacteria bacterium RIFCSPLOWO2_02_FULL_57_10	1
..... Gammaproteobacteria bacterium LSUCC0112	1

.... Chromatiales	34
..... Ectothiorhodospiraceae	15
..... Chromatiaceae	13
..... Chromatiales bacterium	1
..... Granulosicoccaceae	4
..... Halothiobacillaceae bacterium	1
.... Alteromonadales	23
..... Alteromonadaceae	6
..... Psychromonas	10
..... Moritella	5
..... unclassified Idiomarina	2
.... Arenicellales bacterium	1
.... Acidiferrobacterales	2
..... Acidiferrobacterales bacterium	1
..... Acidiferrobacteraceae bacterium	1
.... Porticoccaceae bacterium	1
.... Lysobacterales	2
..... Lysobacter sp.	1
..... Lysobacterales bacterium	1
.... Vibrionales	225
..... Vibrionaceae	224
..... Vibrionales bacterium SWAT-3	1
.... Enterobacterales	11
..... Budviciaceae	6
..... Brenneria	4
...... Brenneria goodwinii	1
...... Brenneria populi	2
.... Brenneria populi subsp. brevivirga	1
...... Brenneria izadpanahii	1
..... Klebsiella pneumoniae	1
.... Aeromonadaceae	6
..... Zobellella	4
..... Oceanisphaera	2
.... Thiotrichales	8
..... Thiotrichales bacterium	1
..... Cocleimonas	6
..... Piscirickettsiaceae bacterium	1
.... Candidatus Competibacteraceae bacterium	1
.... Cardiobacteriales	16
..... Cardiobacteriaceae	7
..... Ostreibacterium oceani	1
..... Ignatzschineria	8
.... Candidatus Competibacterales bacterium	1
... Betaproteobacteria	304
.... Burkholderiales	282
..... Alcaligenaceae	167
..... Burkholderiaceae	10
..... unclassified Burkholderiales	3
..... Burkholderiales genera incertae sedis	3

..... Oxalobacteraceae	40
..... Comamonadaceae	50
..... Sphaerotilaceae	9
.... unclassified Betaproteobacteria	6
.... Nitrosomonadales	4
..... Usitatibacteraceae	3
..... Nitrosomonadaceae bacterium	1
.... Rhodocyclales	8
..... Rhodocyclaceae	4
..... Rhodocyclales bacterium	1
..... Zoogloeaceae	3
.... Betaproteobacteria incertae sedis	4
..... Casimicrobiaceae	3
..... Accumulibacter sp.	1
... Pelomicrobium sp.	1
... Zetaproteobacteria bacterium	1
... Candidatus Lambdaproteobacteria bacterium	1
.. Terrabacteria group	113
... Actinomycetota	6
.... Actinomycetes	2
..... Nocardioides marinus	1
..... Mycobacteroides abscessus subsp. abscessus	1
.... Actinomycetota bacterium	1
.... Acidimicrobiia bacterium	1
.... Thermoleophilia	2
..... Solirubrobacterales bacterium	1
..... Thermoleophilia bacterium	1
... Deinococcota	6
.... Deinococci	5
..... Trueperaceae	3
..... Deinococcales	2
.... Deinococcota bacterium	1
... Bacillota	23
.... Clostridia	18
..... Clostridia bacterium	1
..... Halanaerobiales	14
..... Eubacteriales	3
.... Limnochordales bacterium	1
.... unclassified Bacillota	2
..... Firmicutes bacterium ZCTH02-B6	1
..... Bacillota bacterium	1
.... Virgibacillus halodenitrificans	1
.... Erysipelotrichaceae bacterium	1
... Cyanobacteriota	65
.... Cyanophyceae	59
..... Kaiparowitsia implicata GSE-PSE-MK54-09C	1
..... Leptolyngbyaceae	3
..... Nodosilinea sp. P-1105	1
..... Thermostichales	16

..... Synechococcales	15
..... Nodularia	4
..... Roseofilum	19
.... unclassified Cyanobacteriota	6
..... filamentous cyanobacterium CCP2	1
..... filamentous cyanobacterium CCP1	1
..... Cyanobacteria bacterium J069	1
..... Cyanobacteriota bacterium	1
..... Cyanobacteriota bacterium SKYGB_h_bin112	1
..... Cyanobacteria bacterium UBA11691	1
... Chloroflexota	13
.... Chloroflexia	7
..... Chloroflexia bacterium	1
..... Chloroflexineae	6
.... Candidatus Thermofonsia Clade 3	3
..... unclassified Candidatus Roseilinea	2
..... Candidatus Thermofonsia Clade 3 bacterium	1
.... Chloroflexota bacterium	1
.... Candidatus Limnocylindria bacterium	1
.... Anaerolineales bacterium	1
.. Spirochaetota	29
... Spirochaetia	24
.... Spirochaetales	22
..... Spirochaetaceae	12
..... Sphaerochaetaceae	4
..... Spirochaetales bacterium	1
..... Rectinemataceae bacterium	1
..... Treponema	4
.... Spirochaetia bacterium	1
.... Leptospiraceae bacterium	1
... unclassified Spirochaetota	5
.... Spirochaetota bacterium	1
.... Spirochaetes bacterium GWC2_52_13	1
.... Spirochaetae bacterium HGW-Spirochaetae-8	1
.... Spirochaetae bacterium HGW-Spirochaetae-4	1
.... Spirochaetae bacterium HGW-Spirochaetae-2	1
.. unclassified Bacteria	5
.. Thermodesulfobacteriota	83
... Desulfobacterales	5
.... Desulfosarcinaceae bacterium	1
.... Desulfobacterales bacterium	1
.... Desulfobacteraceae	3
... Desulfobulbales	16
.... Desulfocapsaceae	11
.... Desulfobulbaceae	5
... Desulfovibrionales	56
.... Desulfohalobiaceae	2
.... Desulfovibrionaceae	40
.... Desulfovibrionales bacterium	1

.... Desulfonatronum	4
.... Desulfomicrobiaceae	8
.... Desulfonatronovibrio hydrogenovorans	1
... Thermodesulfobacteriota bacterium	1
... Thermodesulforhabdus norvegica	1
... unclassified Syntrophus (in: bacteria)	3
... Pelovirga terrestris	1
.. Myxococcota	3
... Myxococcia	2
.... Deltaproteobacteria bacterium	1
.... Myxococcales bacterium	1
... Myxococcota bacterium	1
.. Bacteria candidate phyla	16
... unclassified Candidatus Rokuibacteriota	14
... Candidatus Methylomirabilota bacterium	1
... Candidatus Glassbacteria bacterium	1
.. PVC group	36
... Verrucomicrobiota	4
.... Verrucomicrobiia	2
.... Opitutia	2
... Planctomycetota	32
.... Phycisphaerae	6
.... unclassified Planctomycetota	3
.... Planctomycetia	23
.. Nitrospiraceae bacterium	1
.. Deferribacterales	4
... Geovibrio	3
.... Geovibrio ferrireducens	1
.... Geovibrio thiophilus	1
.... Geovibrio sp.	1
... Deferribacteraceae bacterium	1
.. FCB group	5
... Flavobacteriaceae bacterium	1
... Gemmatimonadota	3
.... Gemmatimonadota bacterium	1
.... Gemmatimonadales bacterium	1
.... Longimicrobiales bacterium	1
... Candidatus Neomarinimicrobiota bacterium	1
.. Acidobacteriota	5
... Acidobacteriota bacterium	1
... Acidobacteria bacterium Mor1	1
... Terriglobia	3
.... Candidatus Acidiferrum sp.	1
.... Terriglobales	2
.. Campylobacterota	14
... Campylobacterales	13
... Campylobacterota bacterium	1
.. Chrysiogenaceae	3
... Desulfurispirillum	2

... Chrysiogenes arsenatis	1
.. Deltaproteobacteria	9
... SAR324 cluster bacterium	1
... Bradymonadales	6
... unclassified Deltaproteobacteria	2
.. Nitrospinota/Tectimicrobiota group	5
... Nitrospinaceae bacterium	1
... Candidatus Tectimicrobiota	4
.. Synergistaceae bacterium	1
. uncultured organism	1

**Taxonomy**	**Number of Organisms**
**Archaea**	**203**
. Halobacteriales	201
.. Haloferacaceae	105
... Haloferax	20
... Halohasta	2
... Halorubrum	53
... Halobellus	14
... Salinigranum halophilum	1
... Salinirubrum litoreum	1
... Halalkaliarchaeum desulfuricum	1
... Halonotius	3
... Haloplanus	4
... Halalkalirubrum salinum	1
... Halobaculum sp. MBLA0147	1
... Natronocalculus amylovorans	1
... unclassified Haloferacaceae	2
... Haloquadratum sp. J07HQX50	1
.. Halorussus	4
... Halorussus pelagicus	1
... Halorussus amylolyticus	1
... Halorussus salinisoli	1
... Halorussus marinus	1
.. Haloarculaceae	41
... Haloarcula	31
... Halapricum sp.	1
... Halovenus	2
... Haloarculaceae archaeon	1
... Natronomonas	5
... Salinirubellus salinus	1
.. unclassified Halobacteriales	9
.. Halobacterium	4
... Halobacterium bonnevillei	1
... Halobacterium sp. NMX12-1	1
... Halobacterium hubeiense	1
... Halobacterium jilantaiense	1
.. Natrialbaceae	37

.. Halalkalicoccus sp. CGA53	1
. environmental samples	2
.. uncultured archaeon A07HR67	1
.. uncultured archaeon A07HR60	1

Table S3. Crystallographic data collection and refinement of GpxB and UgpB

Parameters	GpxB-G1P complex	GpxB-G3P complex	UgpB-G1P complex	UgpB-G3P complex
Diffraction data				
Space group	*I*23	*I*23	$P2_12_12$	$P2_12_12$
Unit cell				
a, b, c (Å)	136.28, 136.28, 136.28	136.51, 136.51, 136.51	74.20, 114.91, 46.05	74.45, 115.08, 46.30
α, β, γ (°)	90.0, 90.0, 90.0	90.0, 90.0, 90.0	90.0, 90.0, 90.0	90.0, 90.0, 90.0
Resolution range (Å)	30.47-1.68 (1.72-1.68) *	22.14-2.0 (2.07-2.0)	32.34-1.50 (1.54-1.50)	29.01-1.34 (1.37-1.34)
Redundancy	31.6 (9.8)	3.5 (2.6)	12.4 (8.9)	10.7 (4.0)
Completeness (%)	100.0 (100.0)	98.8 (99.0)	99.0 (90.0)	93.0 (59.2)
R_{merge}**	0.11 (1.22)	0.12 (0.38)	0.09 (1.21)	0.05 (0.65)
$I/\sigma I$	22.0 (1.7)	16.2 (2.3)	18.7 (1.7)	25.4 (1.8)
Refinement statistics				
R-factor	0.15	0.18	0.18	0.18
Free R-factor	0.17	0.19	0.20	0.19
RMSD from ideal geometry				
Bond lengths (Å)	0.006	0.007	0.006	0.006
Bond angles (°)	0.89	0.92	0.86	0.87
Ramachandran plot (%)				
Favored	96.59	95.9	98.04	98.04
Allowed	3.41	4.1	1.96	1.96
Outliers	0	0	0	0
Overall B-factors (Å^2)	24.22	24.47	20.78	19.29

*Numbers in parentheses refer to data in the highest-resolution shell.

** $R_{merge}=\sum_{hkl}\sum_i|I(hkl)_i - \langle I(hkl)\rangle|/\sum_{hkl}\sum_i I(hkl)_i$, where I is the observed intensity, $\langle I(hkl)\rangle$ represents the average intensity, and $I(hkl)_i$ represents the observed intensity of each unique reflection.

Table S4. Volume of the protein binding pockets in the PhnT family.

Protein	PDB code	Ligand	Binding pocket volume ($Å^3$)
HtxB (3.A.1.9.4)	5ME4	Hypophosphite	47
PtxB (3.A.1.9.3)	5JVB	Phosphite	56
	5LQ1	MPn	79
Marine PhnD	5LQ5	Phosphite	61
	5LQ8	MPn	67
PhnD (3.A.1.9.1)	3P7I	2AEP	246
$GpxB^{DSM11874}$	9LF9	G1P	313
	9LFB	G3P	293

Table S5. Volume of the protein binding pockets in the CUT1 family.

Protein	PDB code	Ligand	Binding pocket volume (Å^3)
Trisaccharide-binding protein	2W7Y	A trisaccharide	1453
Trehalose-binding protein	6J9W	Trehalose (disaccharide)	620
Hexitol-binding protein	4RYA	D-mannitol (linear monosaccharide)	220
Glucose-binding protein	5DVI	Glucose (cyclic monosaccharide)	162
UgpB	9LFF	Glycerol-1-phosphate (G1P)	320
	9LFJ	Glycerol-3-phosphate (G3P)	315
GPC Binding Protein	6R1B	Glycerophosphorylcholine (GPC)	796
U3G Binding Protein	7C0F	uridylyl-3’-5’-phosphoguanosine (U3G)	1291

Table S6. List of oligonucleotides, plasmids and bacterial strains used in this study.

Strain or plasmid	Relevant characteristics or sequences	Source or reference
Bacterial strains		
E. coli DH5α	Cloning host for plasmid propagation	Lab collection
E. coli WM3064	Δ*dapA*, auxotrophic for diaminopimelic acid, containing RP4 plasmid transfer machinery	Dehio & Meyer, 1997
E. coli BL21(DE3)	Strain for overexpression of cloned genes in pET vectors	Vazyme Biotech company (China)
Phaeobacter sp. MED193	Wild type	Lab collection
Phaeobacter sp. MED193 *ΔgpxBΔugpB*	*ΔgpxB::Spec*, *ΔugpB::Gm*	This study
Plasmids		
pK18*mobsacB*-GpxB	Suicide vector carrying *gpxB* flanking regions and spectinomycin cassette	This study
pK18*mobsacB*-UgpB	Suicide vector carrying *ugpB* flanking regions and gentamicin cassette	This study
p34S-Gm	Source of the Gm resistance cassette	Lab collection
pHP45-omega	Source of the Spec resistance cassette	Lab collection
pET-22b	Plasmid vector for protein expression in *E. coli*	Novagen
pET-28a	Plasmid vector for protein expression in *E. coli*	Novagen
Primers		
gpxB-UF	ATTCGAGCTCGGTACCCGGGAAGTATTCCGGCATCGTCC	This study
gpxB-UR	CCTTCATCCGCGACCGGGGTATAGGCAAAAAC	This study
gpxB-DF	ACTAAGCTGAATTGTGCTCTACGCCATC	This study
gpxB-DR	TAAAACGACGGCCAGTGCCATTCGCCCCTGTCAA AAC	This study
spec-F	ACCCCGGTCGCGGATGAAGGCACGAACC	This study
spec-R	AGAGCACAATTCAGCTTAGTAAAGCCCTCG	This study
gpxB-CheckF	AAATCCACTGGTGGTGGGTC	This study
gpxB-CheckR	ATTGCGGCAGATTGGCAAAG	This study
ugpB-UF	ATTCGAGCTCGGTACCCGGGACGCTGCTGCAAAAACGTG	This study
ugpB-UR	TGCGTAACATATGAGTTCTCCCCGTTCATC	This study
ugpB-DF	GCCCACCTAAGCACTGCTTAATGATCTG	This study
ugpB-DR	TAAAACGACGGCCAGTGCCAACATGAATACCCACAGCG	This study
Gm-F	GAGAACTCATATGTTACGCAGCAGCAAC	This study
Gm-R	TAAGCAGTGCTTAGGTGGCGGTACTTGG	This study
ugpB-CheckF	GGACTGATACATCGCCTGAC	This study
ugpB-CheckR	CACTTTCCTTCTTCGCCAC	This study
Point-mutation primers		
GpxB^{DSM11874}(Y138A)-F	GCTAATAATTTCCATTTCAGCACCAAAGCTACCATCTGCGG CGG	This study
GpxB^{DSM11874}(Y138A)-R	CCGCCGCAGATGGTAGCTTTGGTGCTGAAATGGAAATTAT TAGC	This study
GpxB^{DSM11874}(S168A)-F	GCCTTAAAACCGCTATTGGCGGTCTGACTGGTAAAGGC	This study
GpxB^{DSM11874}(S168A)-R	GCCTTTACCAGTCAGACCGCCAATAGCGGTTTTAAGGC	This study
GpxB^{DSM11874}(S170A)-F	TGCCTTAAAACCGGCATTGCTGGTCTGACTGGTAAAGGC	This study

GpxB^{DSM11874}(S170A)-R	GCCTTTACCAGTCAGACCAGCAATGCCGGTTTTAAGGCA	This study
GpxB^{DSM11874}(H199A)-F	CCCAGAATGGAATTATCAGCTTTACCAGAAAACACCGGCT CAAAA	This study
GpxB^{DSM11874}(H199A)-R	TTTTGAGCCGGTGTTTTCTGGTAAAGCTGATAATTCCATTC TGGG	This study
GpxB^{DSM11874}(T247A)-F	CGATACCATATCCGGCGGTCGGAAAGGTCTG	This study
GpxB^{DSM11874}(T247A)-R	CAGACCTTTCCGACCGCCGGATATGGTATCG	This study
GpxB^{DSM11874}(R223A)-F	CCCGACTCAGCATGGCATTTAAAACGCTATTTGCAACTGCT	This study
GpxB^{DSM11874}(R223A)-R	AGCAGTTGCAAATAGCGTTTTAAATGCCATGCTGAGTCGG G	This study
UgpB(Y64A)-F	GGTCATCGTTTCGGTAGCATTACCTTTATAAACCGGCACCA CTTTATA	This study
UgpB(Y64A)-R	TATAAAGTGGTGCCGGTTTATAAAGGTAATGCTACCGAAAC GATGACC	This study
UgpB(E88A)-F	CATGGTTGCTGTACCAACTGCAAACACCTGAACAATCTG	This study
UgpB(E88A)-R	CAGATTGTTCAGGTGTTTGCAGTTGGTACAGCAACCATG	This study
UgpB(S143A)-F	ATACCACATAACTGGGGTGGCGCTATTAAAAGGCATGCTC A	This study
UgpB(S143A)-R	TGAGCATGCCTTTTAATAGCGCCACCCCAGTTATGTGGTAT	This study
UgpB(S266A)-F	GCTACCATAATATGCACTAGCATTCATCCACATACCACATT CACC	This study
UgpB(S266A)-R	GGTGAATGTGGTATGTGGATGAATGCTAGTGCATATTATG GTAGC	This study
UgpB(W188A)-F	CCATAACCCAACTCTGCGCGCCAAAGCTAACACCAC	This study
UgpB(W188A)-R	GTGGTGTTAGCTTTGGCGCGCAGAGTTGGGTTATGG	This study
UgpB(W191A)-F	AAATTTTCAACCATAACCGCACTCTGCCAGCCAAAGCTAAC AC	This study
UgpB(W191A)-R	GTGTTAGCTTTGGCTGGCAGAGTGCGGTTATGGTTGAAAA TTT	This study
UgpB(Y342A)-F	CGTCGTAATCGGAACAGCACCGGTTTCCTGATGCCAC	This study
UgpB(Y342A)-R	GTGGCATCAGGAAACCGGTGCTGTTCCGATTACGACG	This study
UgpB(R393A)-F	AGTTCTTCATTAATAACATCGGCAACCTGCACAAAATTACC AAAGCG	This study
UgpB(R393A)-R	CGCTTTGGTAATTTTGTGCAGGTTGCCGATGTTATTAATGA AGAACT	This study

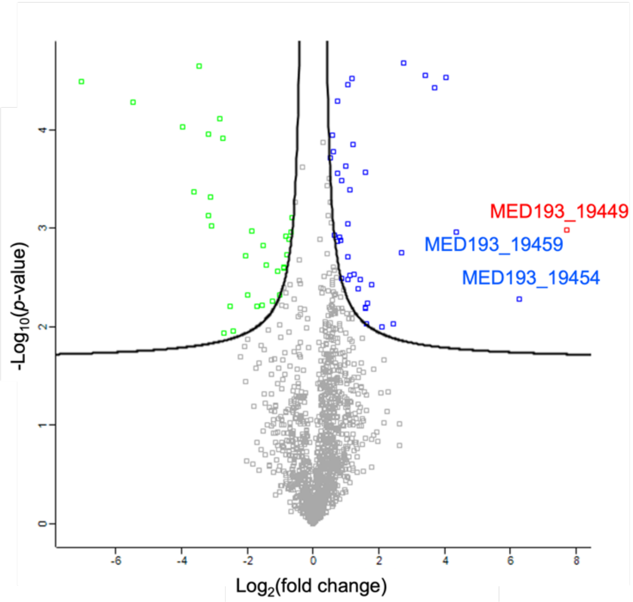

Figure S1. Volcano plots of cellular proteomic data comparing *Phaeobacter* sp. MED193 grown on G3P as sole carbon source to MED193 cultured with glycerol. Scatter points represent proteins. The x-axis is the fold change for the ratio between both growth conditions, and the y-axis is the statistical *p*-value. Green dots represent proteins that are significantly downregulated in the G3P growth condition whereas blue dots represent those proteins that are significantly upregulated (*p*-value <0.01, $\log_2$ fold-change). The GpxB transporter substrate binding protein is highlighted in red.

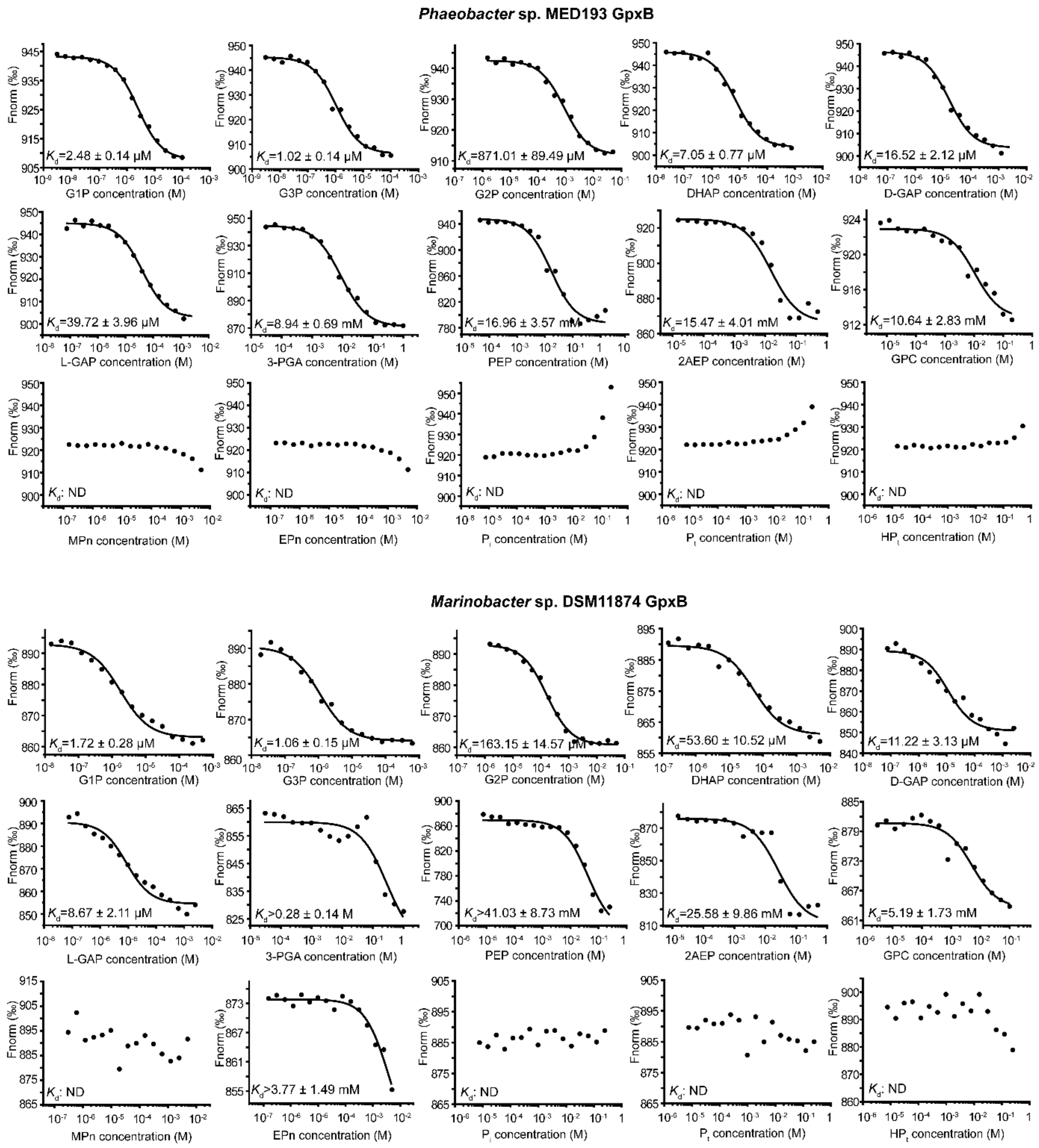

Figure S2. Microscale thermophoresis (MST) binding affinity assays of purified *Phaeobacter* sp. MED193 GpxB (MED193_19449) and GpxB^{DSM11874} (GpxB homolog from *Marinobacter* sp. DSM 11874) on a variety of substrates. The x-axis represents the logarithmic concentration of each serially diluted substrate, and the y-axis represents the normalized fluorescence (Fnorm). ND indicates no binding was detected. G1P, Glycerol-1-phosphate; G3P, Glycerol-3-phosphate; G2P, Glycerol-2-phosphate; DHAP, Dihydroxyacetone phosphate; D-GAP, D-Glyceraldehyde-3-phosphate; L-GAP, L-Glyceraldehyde-3-phosphate; 3-PGA, D-(−)-3-Phosphoglyceric acid; PEP, Phospho(enol)pyruvic acid; 2AEP, 2-Aminoethylphosphonate; GPC, Glycerophosphocholine;

MPn, Methylphosphonate; EPn, Ethylphosphonate; P_i, Inorganic phosphate; P_t, Phosphite; HP_t, Hypophosphite.

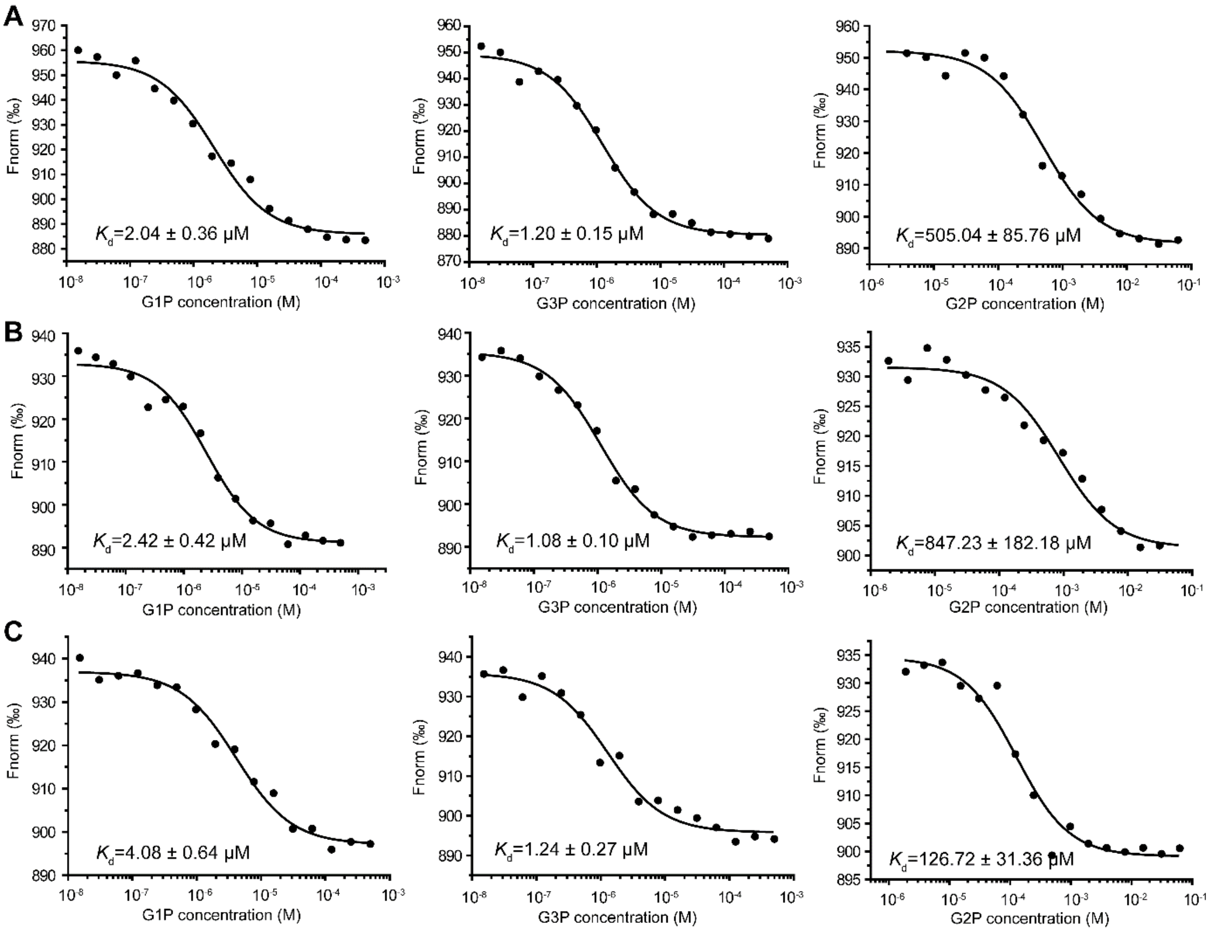

Figure S3. MST binding affinity assays of purified GpxB from several marine bacteria. A) *Sulfitobacter pseudonitzschiae* DSM26824; **B)** *Sulfitobacter* sp. EE-36; **C)** *Sulfitobacter geojensis* sp. EhN01. The x-axis represents the logarithmic concentration of each serially diluted substrate and the y-axis represents the normalized fluorescence (Fnorm).

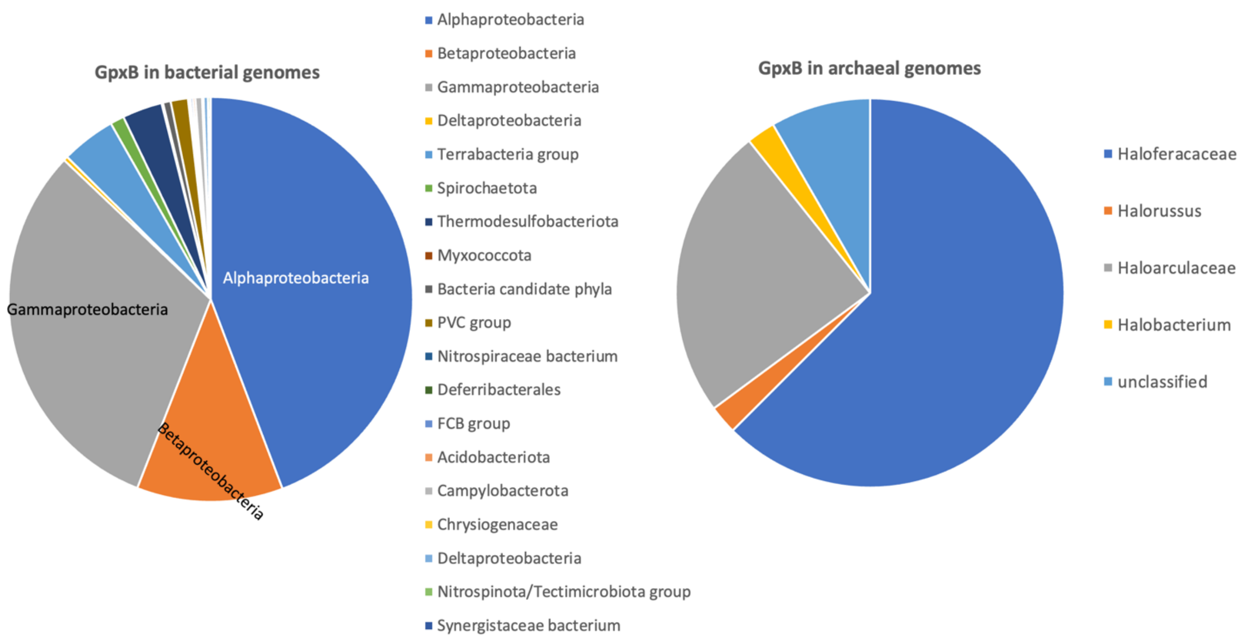

Figure S4. The occurrence of GpxB in bacterial and archaeal genomes. The NCBI nt database was queried with GpxB from *Phaeobacter* sp. MED193, using an e-value -50, sequence coverage >75%.

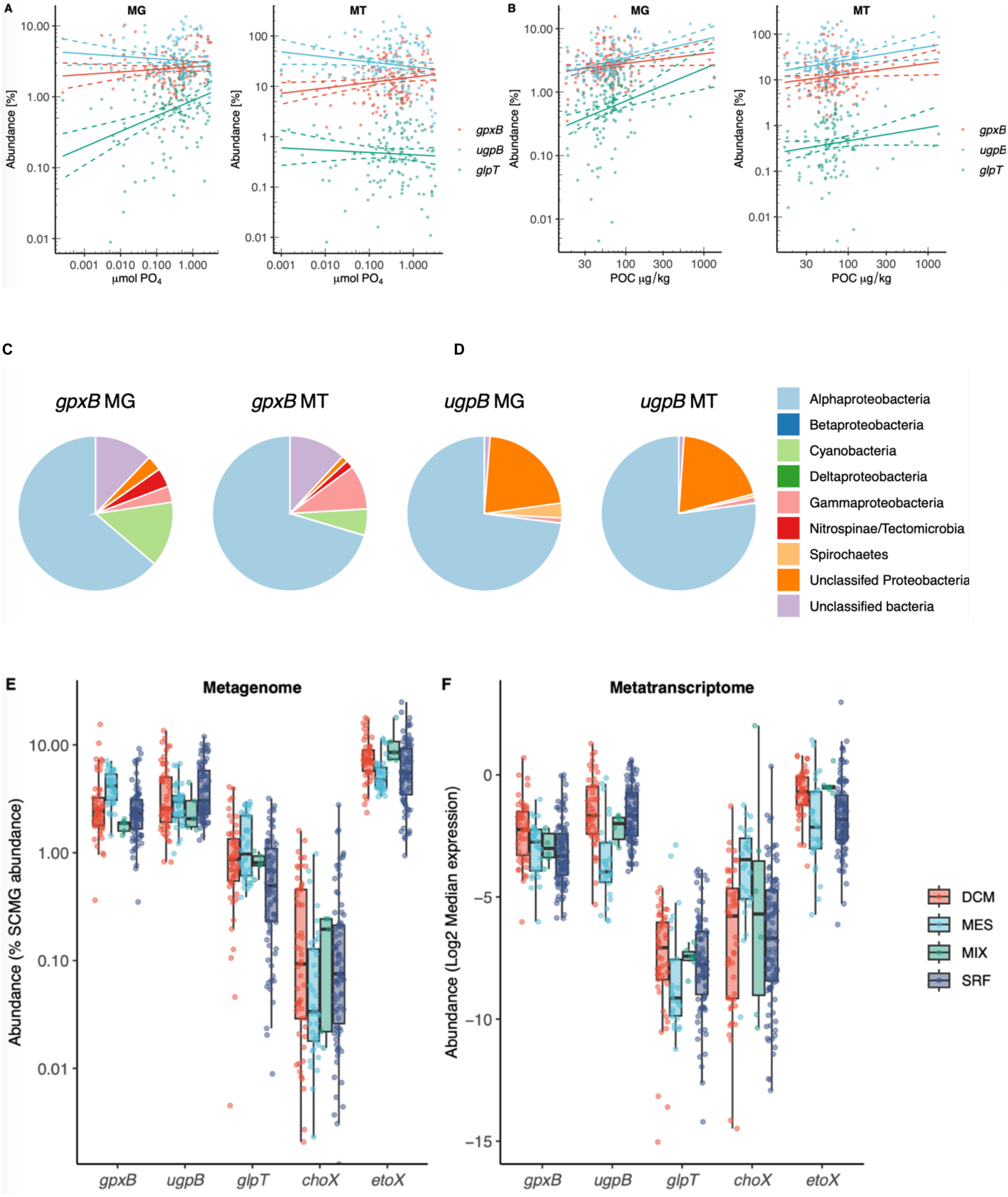

Figure S5. The global distribution and expression of GpxB in the Tara Ocean microbiome. The abundance of the glycerol phosphate substrate binding proteins *gpxB* and *ugpB*, and the major facilitator superfamily glycerol-3-phosphate transporter *glpT*, and their relationship with inorganic phosphate concentrations (**A**) and dissolved organic carbon (**B**), in the metagenome (MG) and metatranscriptome (MT). MG and MT abundance were calculated

as a percentage of the median abundance of 10 single copy marker genes (SCMG) or the equivalent marker transcripts. Linear regressions are shown (solid lines) together with 95% confidence intervals (dashed lines). Taxonomic assignment of sequence abundance normalised across all sites for *gpxB* (**C**) and *ugpB* (**D**). Metagenomic (**E**) and metagenomic (**F**) abundance of transporters/substrate binding proteins by sampling site type. Metagenomic abundance was calculated as in panels **A** and **B**. Metatranscriptomic abundance was calculated by a $\log_2$ transformation of transcript abundance normalised to the median abundance of the same 10 single copy marker transcripts. The abundance of other lipid headgroup transporters is included for comparison – *choX* (choline) and *etoX* (ethanolamine). DCM = deep chlorophyll maximum, MES = mesopelagic, MIX = wind mixed layer, SRF = surface.

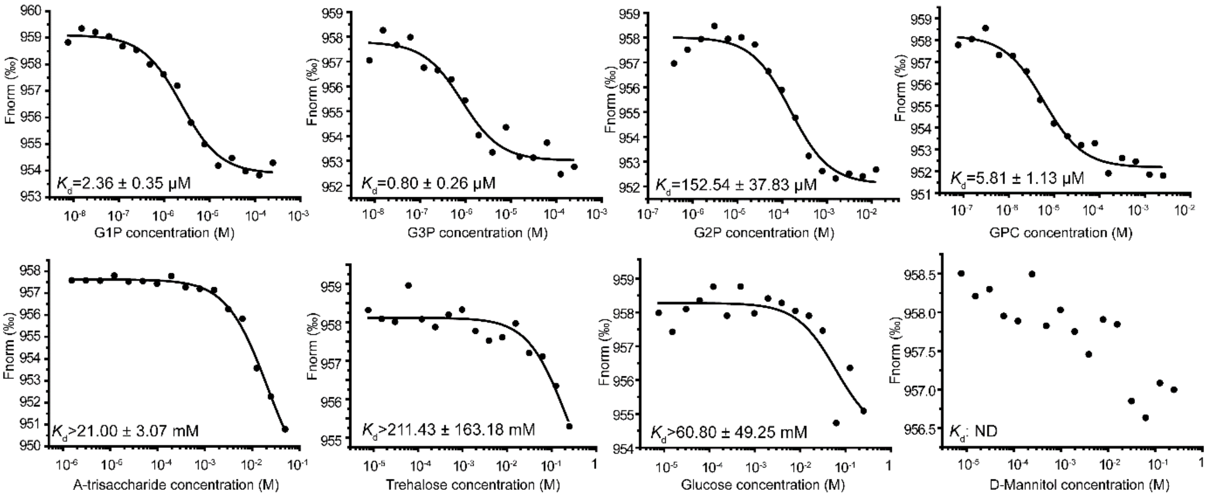

Figure S6. MST binding affinity assays of purified *Phaeobacter* sp. MED193 UgpB (MED193_07903) on various substrates. The x-axis represents the logarithmic concentration of each serially diluted substrate and the y-axis represents the normalized fluorescence (Fnorm). ND indicates no binding was detected.

A

Protein	G1P K_d (μM)	G3P K_d (μM)
WT	1.72 ± 0.28	1.06 ± 0.15
Y138A	153.36 ± 17.93	39.78 ± 2.83
S168A	8.75 ± 0.61	4.44 ± 0.27
S170A	3.09 ± 0.42	1.74 ± 0.31
H199A	171.28 ± 17.98	52.52 ± 4.19
T247A	92.07 ± 8.89	68.04 ± 6.13
R223A	15.43 ± 2.00	57.00 ± 4.88

B

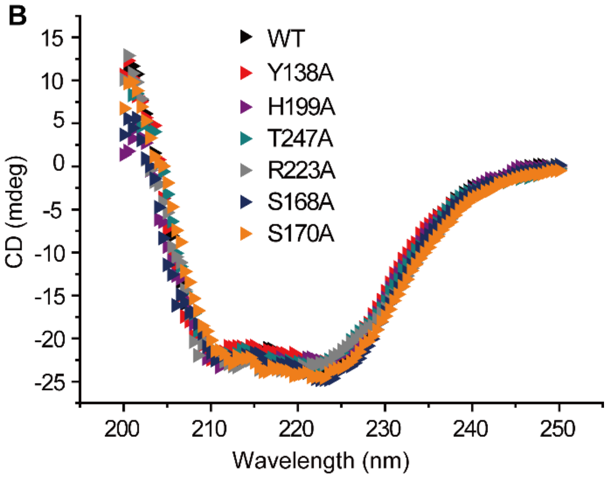

Figure S7. Characterisation of site-directed mutants of *Marinobacter* sp. DSM 11874 GpxB^{DSM11874}. A) MST binding affinity assays of GpxB^{DSM11874} mutants on G1P and G3P; **B)** Circular-dichroism (CD) spectra of GpxB^{DSM11874} mutants.

A

Protein	G1P K_d (μM)	G3P K_d (μM)
WT	2.36 ± 0.35	0.80 ± 0.26
Y64A	ND	ND
E88A	ND	ND
S143A	ND	ND
S266A	ND	ND
W188A	2793.48 ± 791.02	332.13 ± 111.73
W191A	1181.83 ± 318.00	38.89 ± 11.06
Y342A	ND	ND
R393A	ND	ND

B

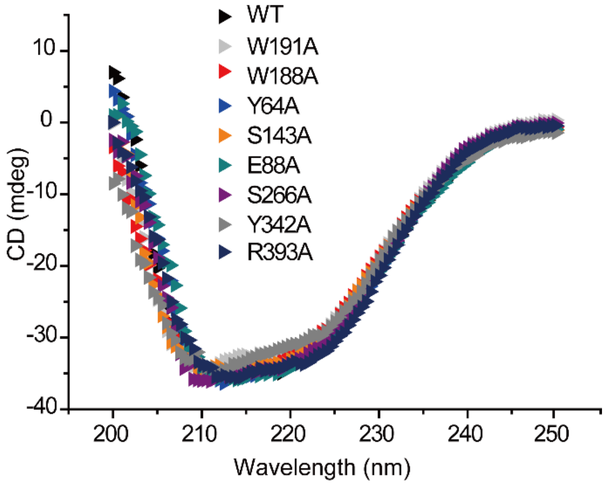

Figure S8. Characterisation of site-directed mutants of *Phaeobacter* sp. MED193 UgpB. A) MST binding affinity assays of UgpB mutants on G1P and G3P; **B)** CD spectra of UgpB mutants. ND indicates no binding was detected.

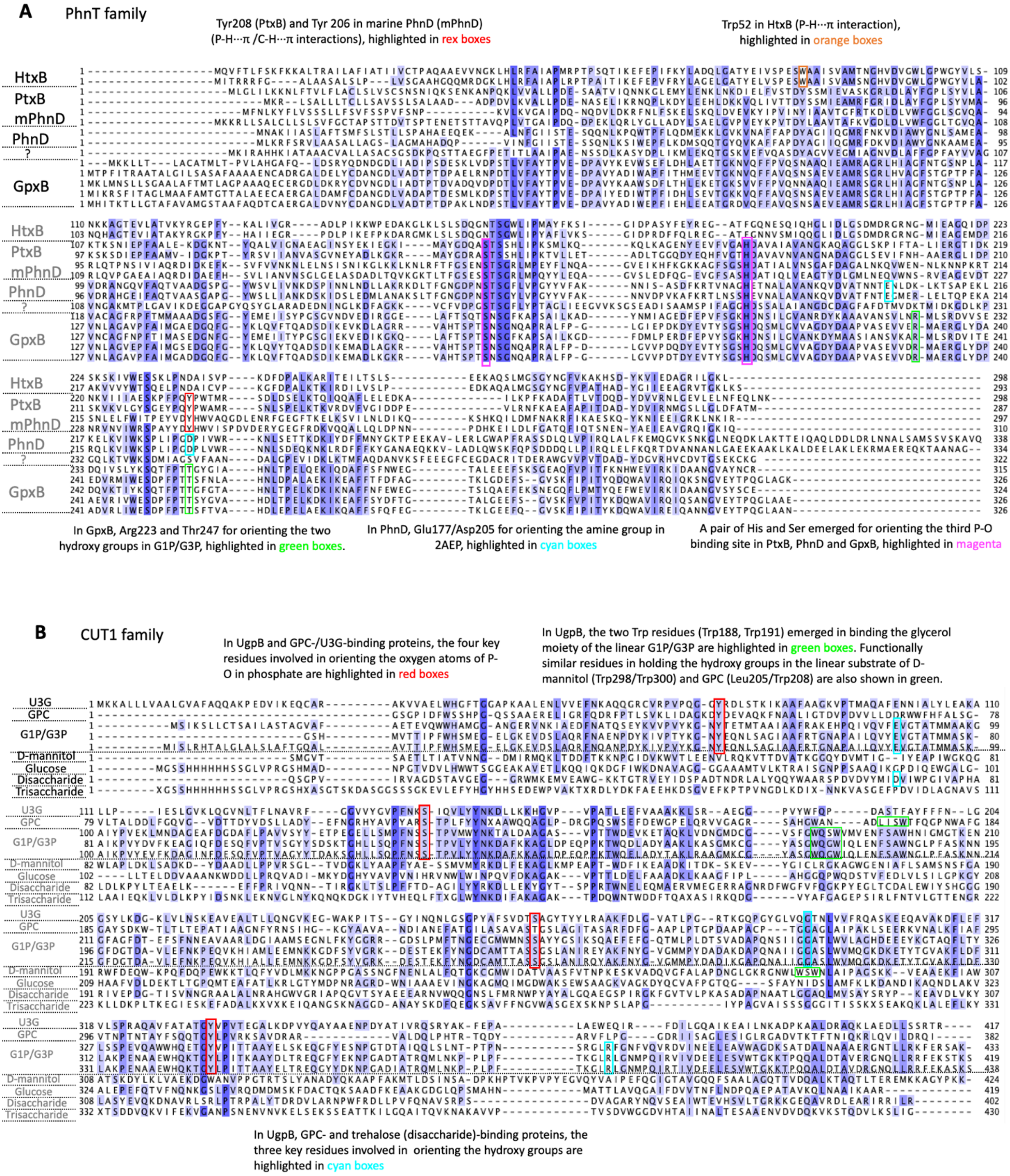

Figure S9. Multiple sequence alignment of A) PhnT family members (including GpxB) and B) CUT1 family proteins (including UgpB).

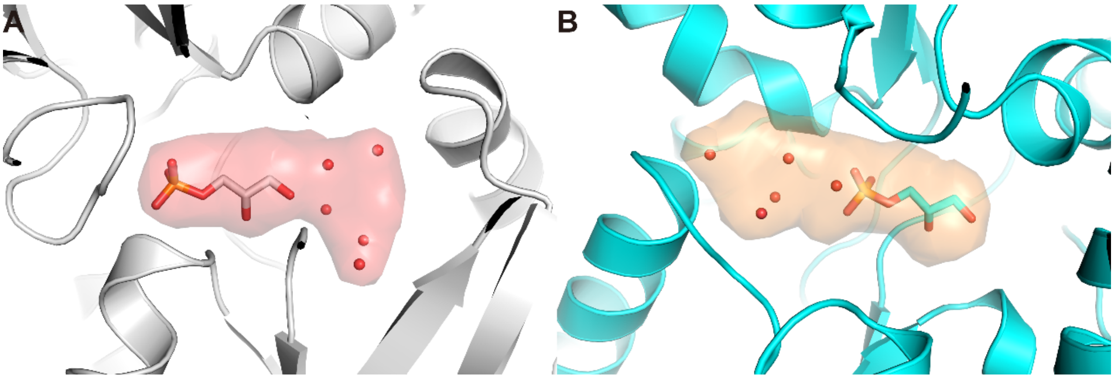

Figure S10. A comparison of the binding site cavity in GpxB^{DSM11874} and UgpB.

A) The G1P binding site of the GpxB^{DSM11874}-G1P complex. The G1P molecule is shown as grey sticks, and five water molecules occupying the binding cavity are represented as red spheres. The binding site cavity in GpxB is displayed as a red semi-transparent surface.

B) The G1P binding site of the UgpB-G1P complex. The G1P molecule is shown as cyan sticks and five water molecules occupying the binding cavity are represented as red spheres. The binding site cavity in UgpB is displayed as an orange semi-transparent surface. The cavity size was calculated using PyVOL.
